# Protease-catalysed Direct Asymmetric Mannich Reaction in Organic Solvent

**DOI:** 10.1038/srep00761

**Published:** 2012-10-23

**Authors:** Yang Xue, Ling-Po Li, Yan-Hong He, Zhi Guan

**Affiliations:** 1 School of Chemistry and Chemical Engineering, Southwest University, Chongqing, 400715, P. R. China

## Abstract

We reported the first enzyme-catalysed, direct, three-component asymmetric Mannich reaction using protease type XIV from *Streptomyces griseus* (SGP) in acetonitrile. Yields of up to 92% with enantioselectivities of up to 88% e.e. and diastereoselectivities of up to 92:8 (*syn*:*anti*) were achieved under the optimised conditions. This enzyme's catalytic promiscuity expands the application of this biocatalyst and provides a potential alternative method for asymmetric Mannich reactions.

Enzyme catalytic promiscuity, in which a single active site of a given enzyme can catalyse different chemical transformations of natural or non-natural substrates^1^, has received widespread attention as more catalytic promiscuities of existing enzymes have been discovered. In fact, all enzymes are thought to have evolved as a result of promiscuous activities in the primordial, ancient enzymes. The relatively few rudimentary ancestral generalist enzymes acted on multiple substrates to afford a wider range of metabolic capabilities. The increased catalytic specificity and selectivity are thought to be a result of divergence and evolution^2^,^3^,^4^,^5^,^6^,^7^. Thus, enzyme catalytic promiscuity is a key factor in the evolution of new enzyme functions. Moreover, promiscuous activity does not normally affect an organism if the promiscuous reaction does not affect the rate of the native activity or if the substrate for the promiscuous reaction is not natural to the enzyme. So there is no selective pressure to remove the promiscuous reaction. Catalytically promiscuous behaviour is often hidden behind a native catalytic transformation and only visible under non-natural conditions^2^,^6^,^8^,^9^. Therefore, investigations of enzyme catalytic promiscuity not only gain fundamental knowledge about enzyme/substrate interactions and the evolution of new enzymes but also help to better understand metabolic pathways for the biosynthesis of secondary metabolites. Furthermore, enzyme catalytic promiscuity could expand the application of biocatalysts and provide a useful avenue in organic synthesis. To date, no general methods are available to profile enzyme catalytic promiscuity and it is necessary to evaluate enzyme catalytic promiscuity for existing enzymes case by case.

Many examples of enzyme catalytic promiscuity in organic synthesis have been reported recently^10^. Several enzymes have been used in asymmetric syntheses in organic solvents, including asymmetric aldol reactions^11^,^12^, C-C Michael additions^13^, *β*-lactam opening^14^, the asymmetric synthesis of *α*-aminonitrile amides^15^ and the preparation of chiral epoxides^16^. An enzyme-catalysed asymmetric Mannich reaction has not yet been reported, even though some groups have reported enzyme-catalysed Mannich reactions without enantioselectivity^17^,^18^,^19^,^20^. The asymmetric Mannich reaction is a powerful synthetic strategy to prepare chiral β-amino ketones and aldehydes with perfect atom economy through the loss of a molecule of water and the reaction products are versatile intermediates in the synthesis of chiral amines^21^. This characteristic makes it important to develop an enzyme-catalysed asymmetric Mannich reaction as a more sustainable complement to chemical catalysis. In this context, we investigated the asymmetric one-pot Mannich reaction catalysed by the protease type XIV from *Streptomyces griseus* (SGP) without the need for additional cofactors or special equipment.

## Results

### Control experiments

Initially, the Mannich reaction of cyclohexanone, 4-nitrobenzaldehyde and aniline was used as a model reaction. We found that SGP was able to catalyse the model reaction in MeCN in the presence of water, which gave the product in a moderate yield of 66% with a good enantioselectivity of 82% e.e. for the *syn*-isomer and 85:15 dr (*syn*:*anti*) (Table 1, entry 1). To verify the specific catalytic effect of SGP on the Mannich reaction, some control experiments were performed (Table 1, entries 2–12). In the absence of the enzyme, the Mannich product was only obtained in a yield of 28% (Table 1, entry 2), indicating that SGP indeed had a catalytic effect on the Mannich reaction. To further confirm the effect of background and SGP on the reaction, we profiled both the blank reaction and the SGP-catalysed model Mannich reaction (for details, please see Supplementary Tables S1–S2). Next, the albumins from chicken egg white and from bovine serum were used separately in the model reaction as non-enzyme proteins, which produced the Mannich products in 23% yield with 0% e.e. and 21% yield with 7% e.e., respectively (Table 1, entries 3 and 4). These reactions excluded the possibility of protein catalysis, meaning that catalysis was not simply a result of the amino acid residues on the surface of the protein. Furthermore, urea-denatured SGP was used to catalyse the model reaction, which gave a high yield of 87% with only 8% e.e. (Table 1, entry 5). The same amount of urea was then used to catalyse the reaction, but produced a result nearly indential to the blank, proving that urea alone did not catalyse this transformation (Table 1, entry 6). These results show that the urea-denatured SGP still had catalytic activity towards the Mannich reaction, but it almost completely lost its enantioselectivity. Metal-denatured SGP was also used to catalyse the model reaction to determine whether the metal ion could disrupt the bonds that hold the enzyme together and cause the enzyme to undergo a conformational change, disrupt the active site and ultimately denature the enzyme. SGP was pretreated with Cu^2+^ and Ag^+^ at different concentrations. A low concentration (2.5 mM) of Cu^2+^ or Ag^+^ did not have an obvious effect on the activity and selectivity of SGP towards the Mannich reaction (Table 1, entries 7 and 10), while a moderate concentration (25 mM) of Cu^2+^ or Ag^+^ caused a slight decrease in enantioselectivity (Table 1, entries 8 and 11). A higher concentration (250 mM) of Cu^2+^ or Ag^+^ almost completely destroyed the selectivity of SGP in the model Mannich reaction (Table 1, entries 9 and 12). From the above control experiments with urea or metal ion-denatured SGP, we determined that the denatured SGP still exhibited catalytic activity in the Mannich reaction, but it lost nearly all of its stereoselectivity, indicating that the specific natural fold of SGP is responsible for its stereoselectivity in Mannich reactions.

### Optimisation of reaction conditions

Next, we explored the effects of different solvents on the SGP-catalysed model Mannich reaction (Table 2). The reaction medium played an important role in this enzymatic reaction. The highest selectivity of 82% e.e. for the *syn* isomer (85:15 dr, *syn*:*anti*) was obtained with a moderate yield in MeCN (Table 2, entry 1), whereas water gave the highest yield of 76% with low selectivity (Table 2, entry 12). The reaction in DMF gave the lowest yield of 16% (Table 2, entry 11) and the reaction in DMSO provided the lowest enantioselectivity of only 6% e.e. with reversed diastereoselectivity (36:64 dr, *syn*:*anti*) (Table 2, entry 15). Generally, no clear correlation was observed between solvent polarity and enzyme activity or selectivity. Thus, to optimise selectivity, we selected MeCN as the solvent for the SGP-catalysed Mannich reaction.

Water content, pH, molar ratio of substrates and temperature are also important factors in enzymatic reactions^22^,^23^,^24^,^25^ and their influence on the SGP-catalysed model Mannich reaction was investigated next. The optimised reaction conditions were found to consist of the following: a water content of 0.1 [H_2_O/(H_2_O + MeCN), v/v], a molar ratio of 15:1 (cyclohexanone to 4-nitrobenzaldehyde) and a temperature of 30°C. The effect of pH was investigated using a phosphate buffer (NaH_2_PO_4_-Na_2_HPO_4_, 0.2 M, pH 4.60–7.18) to replace the optimised water content in the reaction system [buffer/(buffer + MeCN) = 0.1, v/v], but yields and selectivities were not improved in the presence of buffer. Therefore, we ultimately chose MeCN/H_2_O as the reaction medium for the SGP-catalysed Mannich reaction. Finally, the time course of the SGP-catalysed model Mannich reaction was also investigated with respect to the above-mentioned variables in this section. (for details, please see Supplementary Tables S3–S7).

### Investigation of substrate scope

To investigate the generality and scope of this biocatalytic promiscuity, several other substrates were tested to expand upon this novel SGP-catalysed direct asymmetric Mannich reaction. Various substituted aromatic aldehydes performed well in the reaction. Generally, the aromatic aldehydes containing an electron-withdrawing group exhibited better enantioselectivity and diastereoselectivity than those containing an electron-donating group (Table 3, entries 1–9). Substituent position also had some impact on the selectivity of the reaction. For instance, 3-fluorobenzaldehyde yielded better diastereoselectivity and enantioselectivity than 4-fluorobenzaldehyde (Table 3, entries 5 and 6). In addition to aniline, various substituted arylamines were also used as substrates (Table 3, entries 10–15). Among them, the reaction of 3-bromobenzenamine with 4-nitrobenzaldehyde and cyclohexanone gave the best enantioselectivity at 88% e.e. and the best diastereoselectivity of 92:8 (*syn*:*anti*) (Table 3, entry 10). Furthermore, it was promising that the heteroatom-containing cycloketone was also available as a substrate in this biocatalytic process. The reactions of tetrahydrothiopyran-4-one with aniline and either 4-chlorobenzaldehyde or 4-(trifluoromethyl)benzaldehyde gave satisfactory yields and moderate e.e. values (Table 3, entries 16 and 17). In addition, linear chain ketones could be used as substrates. Acetone, for example, gave a low yield of 22% and no enantioselectivity when reacting with 4-nitrobenzaldehyde and 4-methoxybenzenamine. In almost all cases, the aldol reaction products were observed as side products. Moreover, SGP showed different degrees of enantioselectivity for *syn-*isomers, but low or no enantioselectivity for *anti-*isomers, potentially indicating that SGP has a specific selectivity for the Mannich reaction.

## Discussion

In conclusion, we reported a novel SGP-catalysed, direct, three-component asymmetric Mannich reaction. The control experiments with the denatured enzyme and non-enzyme proteins indicated that the specific natural fold of SGP was responsible for its stereoselectivity in the Mannich reaction. The influence of several factors, including solvent, water content, pH, molar ratio of substrates and temperature, was investigated. A wide range of substrates were accepted by the enzyme and yields of up to 92%, enantioselectivities of up to 88% e.e. and diastereoselectivities of up to 92:8 dr were achieved. As an example of enzyme catalytic promiscuity, this work broadens the scope of SGP-catalysed transformations. Exploring the untapped catalytic potential of natural enzymes could give useful insights into enzyme evolution.

## Methods

### Materials

The protease type XIV from *Streptomyces griseus* was purchased from Sigma-Aldrich (catalogue no. P5147, 6.1 U/mg). One unit will hydrolyse casein to produce colour equivalent to 1.0 μmole (181 μg) of tyrosine per min at pH 7.5 at 37°C (colour by Folin-Ciocalteu reagent). Unless otherwise noted, all reagents were obtained from commercial suppliers and used without further purification.

### General procedure for the SGP-catalysed Mannich reaction

SGP (50 mg) was added to a round-bottom flask containing an aromatic aldehyde (0.5 mmol), an arylamine (0.55 mmol), a ketone (7.5 mmol), MeCN (0.9 mL) and deionised water (0.1 mL). The resultant mixture was stirred at 30°C for the specified reaction time and monitored by TLC on Haiyang GF 254 silica gel plates. The reaction was terminated by filtering the enzyme. The filter cake was washed with ethyl acetate (10 mL). Then, the filtrate was concentrated in vacuo. The residue was purified by flash column chromatography (ethyl acetate/petroleum ether) to give the product.

## Author Contributions

Guan and He designed and supervised the research. Xue and Li performed the experiments. Xue analysed data and drafted the paper. Guan and He reviewed and edited the manuscript. All the authors discussed the results and contributed extensively to the work presented in this paper.

## Supplementary Material

Supplementary Information Supplementary information for the paper Protease-catalysed Direct Asymmetric Mannich Reaction in Organic Solvent

## Figures and Tables

**Table 1:** Control experiments for the SGP-catalysed Mannich reaction^a^

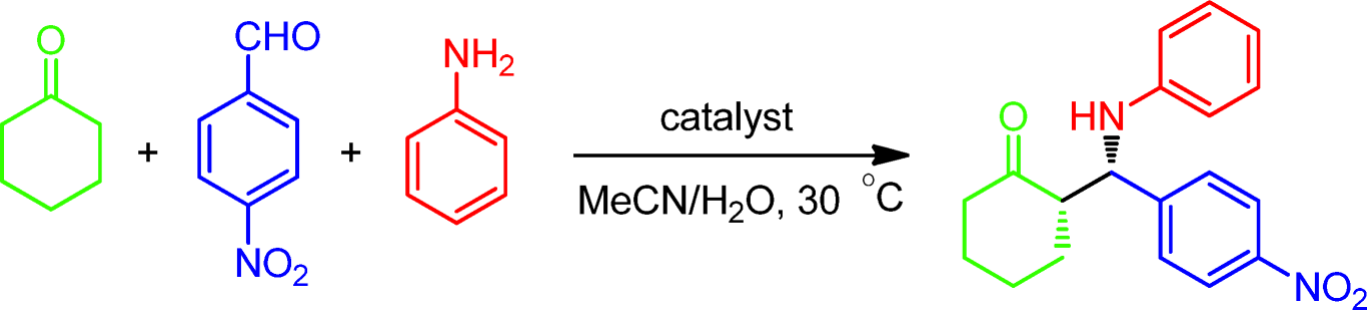				
Entry	Catalyst	Yield (%)^b^	dr (*syn*:*anti*)^c^	e.e. (*syn*) (%)^d^
1	protease type XIV from *Streptomyces griseus* (SGP)	66	85:15	82
2	no enzyme	28	41:59	0
3	albumin from chicken egg white	23	46:54	0
4	albumin from bovine serum	21	58:42	7
5^e^	SGP denatured with urea	87	48:52	8
6^f^	urea	28	39:61	0
7^g^	SGP pretreated with 2.5 mM Cu^2+^	62	88:12	82
8^g^	SGP pretreated with 25 mM Cu^2+^	61	87:13	77
9^g^	SGP pretreated with 250 mM Cu^2+^	51	43:57	9
10^g^	SGP pretreated with 2.5 mM Ag^+^	60	88:12	82
11^g^	SGP pretreated with 25 mM Ag^+^	65	82:18	74
12^g^	SGP pretreated with 250 mM Ag^+^	67	38:62	8

^a^Unless otherwise noted, the reaction conditions were as follows: 4-nitrobenzaldehyde (0.5 mmol), aniline (0.55 mmol), cyclohexanone (5 mmol), deionised water (0.10 mL), MeCN (0.9 mL) and catalyst (50 mg) at 30°C for 96 h.

^b^Yield of the isolated product after silica gel chromatography.

^c^The dr was the *syn*:*anti* ratio, as determined by chiral HPLC analysis.

^d^e.e. value of the *syn*-isomer, determined by HPLC using a chiralpak AD-H column; the absolute configuration was assigned by comparison with the literature (for details, please see the Supplementary Information).

^e^SGP (50 mg) in urea solution (0.42 M) [urea (50 mg) in deionised water (2 mL)] was stirred at 100°C for 24 h and then water was removed under reduced pressure before use.

^f^Urea (50 mg) was used instead of SGP.

^g^The mixture of SGP (50 mg), deionised water (1 mL) and the specified amount of CuSO_4_ (for entries 7–9) or AgNO_3 _(for entries 10–12) was stirred at 30°C for 24 h and then water was removed under reduced pressure before use.

**Table 2:** Influence of solvents on the SGP-catalysed Mannich reaction^a^

Entry	Solvent	Yield (%)^b^	dr (*syn*:*anti*)^c^	e.e. (*syn*) (%)^d^
1	MeCN	66	85:15	82
2	CH_2_Cl_2_	68	74:26	73
3	isopropyl ether	74	69:31	70
4	THF	64	63:37	69
5	ethyl acetate	65	69:31	68
6	MTBE	73	62:38	66
7	butyl acetate	72	67:33	66
8	cyclohexane	71	65:35	64
9	isopropanol	54	62:38	63
10	EtOH	53	72:28	61
11	DMF	16	41:59	51
12	H_2_O	76	58:42	49
13	MeOH	61	63:37	49
14	1,4-dioxane	51	52:48	33
15	DMSO	23	36:64	6

^a^Reaction conditions: a mixture of 4-nitrobenzaldehyde (0.5 mmol), aniline (0.55 mmol), cyclohexanone (5 mmol), deionised water (0.10 mL), solvent (0.9 mL) and SGP (50 mg) was stirred at 30°C for 96 h.

^b^Yield of the isolated product after silica gel chromatography.

^c^Determined by chiral HPLC analysis.

^d^e.e. value of the *syn*-isomer, determined by chiral HPLC using a chiralpak AD-H column.

**Table 3:** Investigation of substrate scope for the SGP-catalysed asymmetric Mannich reaction^a^

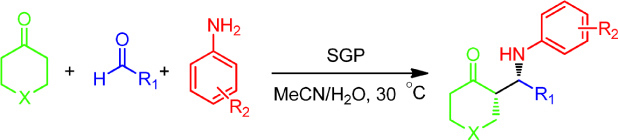								
Entry	X	R_1_	R_2_	Product No.	Time (h)	Yield (%)^b^	dr (*syn*:*anti*)^c^	e.e. (*syn*) (%)^d^
1	CH_2_	4-NO_2_C_6_H_4_	H	4a	96	64	88:12	83
2	CH_2_	4-CF_3_C_6_H_4_	H	4b	94	73	81:19	78
3	CH_2_	4-BrC_6_H_4_	H	4c	94	65	74:26	76
4	CH_2_	4-ClC_6_H_4_	H	4d	120	92	78:22	75
5	CH_2_	3-FC_6_H_4_	H	4e	94	72	70:30	74
6	CH_2_	4-FC_6_H_4_	H	4f	94	66	66:34	64
7	CH_2_	4-CNC_6_H_4_	H	4g	120	61	58:42	68
8	CH_2_	C_6_H_5_	H	4h	100	62	60:40	61
9	CH_2_	4-CH_3_C_6_H_4_	H	4i	120	68	40:60	33
10	CH_2_	4-NO_2_C_6_H_4_	3-Br	4j	144	24	92:8	88
11	CH_2_	4-NO_2_C_6_H_4_	3-CH_3_	4k	123	54	91:9	83
12	CH_2_	4-NO_2_C_6_H_4_	4-Cl	4l	144	47	89:11	83
13	CH_2_	4-NO_2_C_6_H_4_	4-CH_3_	4m	120	81	90:10	82
14	CH_2_	4-NO_2_C_6_H_4_	4-OCH_3_	4n	117	71	72:28	72
15	CH_2_	4-BrC_6_H_4_	4-OCH_3_	4o	165	66	52:48	40
16^e^	S	4-ClC_6_H_4_	H	4p	168	81	57:43	58
17^e^	S	4-CF_3_C_6_H_4_	H	4q	142	80	44:56	52

^a^Reaction conditions: a mixture of aromatic aldehyde (0.5 mmol), arylamine (0.55 mmol), ketone (7.5 mmol), deionised water (0.10 mL), MeCN (0.9 mL) and SGP (50 mg) was stirred at 30°C.

^b^Yield of the isolated product after silica gel chromatography.

^c^Determined by chiral HPLC analysis.

^d^e.e. value of the *syn*-isomer, determined by chiral HPLC; the absolute configuration was assigned by comparison with the literature (for details, please see the Supplementary Information).

^e^tetrahydrothiopyran-4-one (1 mmol).
