# Supplementary material for: Protease-catalysed Direct Asymmetric Mannich Reaction in Organic Solvent
Source: Sci Rep. 2012 Oct 23;2:761. doi: 10.1038/srep00761 (PMC3478581; doi:10.1038/srep00761)
Supplement: Supplementary Information — for the paper Protease-catalysed Direct Asymmetric Mannich Reaction in Organic Solvent [file srep00761-s1.pdf]

Supplementary information for the paper

Protease-catalysed Direct Asymmetric Mannich Reaction in Organic Solvent

Yang Xue, Ling-Po Li, Yan-Hong He* & Zhi Guan*

School of Chemistry and Chemical Engineering, Southwest University, Chongqing, 400715, P. R. China

Fax: +86-23-68254091; e-mail: guanzhi@swu.edu.cn for Z. Guan, heyh@swu.edu.cn for Y-H He

Contents

1. Reaction profiles of the SGP-catalysed Mannich reaction and the blank reaction

2. Optimisation of reaction conditions for the SGP-catalysed Mannich reaction

3. Characterization of the Mannich products

4. ^{1}H NMR, ^{13}C NMR and HPLC spectra for the Mannich products **4a-4r**

5. HPLC spectra for reaction profiles of the SGP-catalysed the Mannich reaction and the blank reaction

6. References

1. Reaction profiles of the SGP-catalysed Mannich reaction and the blank reaction.

Supplementary Table S1. Reaction profile of the blank reaction[a] .

Entry	Time (h)	Yield (%)[b]	dr (*syn*:*anti*)[c]
1	6	no observed	--
2	12	trace	--
3	18	2	53:47
4	24	9	44:56
5	36	11	47:53
6	48	16	41:59
7	60	18	39:61
8	72	20	39:61
9	96	27	41:59

[a]Reaction conditions: a mixture of 4-nitrobenzaldehyde (0.5 mmol), aniline (0.55 mmol), cyclohexanone (5 mmol), deionised water (0.10 mL), MeCN (0.9 mL) was stirred at 30 ˚C for 6-96 h. [b]Yield of the isolated product after silica gel chromatography. [c]Determined by chiral HPLC analysis.

Supplementary Table S2. Reaction profile of the SGP-catalysed Mannich reaction[a].

Entry	Time (h)	Yield (%)[b]	dr (*syn*:*anti*)[c]	e.e. (*syn*) (%)[d]
1	6	8	96:4	82
2	12	20	91:9	82
3	18	28	93:7	83
4	24	31	92:8	80
5	36	46	91:9	83
6	48	52	91:9	84
7	60	57	91:9	83
8	72	59	91:9	84
9	96	64	91:9	84

[a]Reaction conditions: a mixture of 4-nitrobenzaldehyde (0.5 mmol), aniline (0.55 mmol), cyclohexanone (5 mmol), deionised water (0.10 mL), MeCN (0.9 mL) and SGP (50 mg) was stirred at 30 ˚C for 6-96 h. [b]Yield of the isolated product after silica gel chromatography. [c]Determined by chiral HPLC analysis. [d]e.e. value of the *syn*-isomer, determined by chiral HPLC using a chiralpak AD-H column.

2. Optimisation of reaction conditions for the SGP-catalysed Mannich reaction.

Supplementary Table S3. Influence of water content on the SGP-catalysed Mannich reaction[a].

Entry	Water content (%)	Yield (%)[b]	dr (*syn*:*anti*)[c]	e.e. (*syn*) (%)[d]
1	0	30	70:30	42
2	5	67	80:20	73
3	10	66	85:15	82
4	15	68	87:13	81
5	20	73	86:14	80
6	25	77	83:17	77
7	30	72	82:18	75
8	35	70	78:22	73

[a]Reaction conditions: a mixture of 4-nitrobenzaldehyde (0.5 mmol), aniline (0.55 mmol), cyclohexanone (5 mmol), deionised water [0-0.35, H_2O / (H_2O + MeCN), v/v], MeCN (H_2O + MeCN = 1mL) and SGP (50 mg) was stirred at 30 ˚C for 96 h. [b]Yield of the isolated product after silica gel chromatography. [c]Determined by chiral HPLC analysis. [d]e.e. value of the *syn*-isomer, determined by chiral HPLC using a chiralpak AD-H column.

Supplementary Table S4. Effect of pH on the SGP-catalysed Mannich Reaction[a].

Entry	pH	Yield (%)[b]	dr (*syn*:*anti*)[c]	e.e. (*syn*) (%)[d]
1	4.60	85	79:21	78

2	5.55	70	85:15	80
3	6.45	68	87:13	81
4	7.00	69	86:14	82
5	7.18	59	89:11	82

[a]Reaction conditions: a mixture of 4-nitrobenzaldehyde (0.5 mmol), aniline (0.55 mmol), cyclohexanone (5 mmol), a phosphate buffer (NaH_2PO_4-Na_2HPO_4, 0.2 M, pH 4.60-7.18, 0.1 mL), MeCN (0.9 mL) and SGP (50 mg) was stirred at 30 ˚C for 96 h. [b]Yield of the isolated product after silica gel chromatography. [c]Determined by chiral HPLC analysis. [d] e.e. value of the *syn*-isomer, determined by chiral HPLC using a chiralpak AD-H column.

Supplementary Table S5. Effect of molar ratio of substrates on the SGP-catalysed Mannich reaction[a].

Entry	Molar ratio[b]	Yield (%)[c]	dr (*syn*:*anti*)[d]	e.e. (*syn*) (%)[e]
1	1:1	51	62:38	62
2	5:1	72	76:24	78
3	10:1	66	85:15	82
4	15:1	64	88:12	83
5	20:1	62	88:12	82

[a]Reaction conditions: a mixture of 4-nitrobenzaldehyde (0.5 mmol), aniline (0.55 mmol), cyclohexanone (0.5-10 mmol), deionised water (0.10 mL), MeCN (0.9 mL) and SGP (50 mg) was stirred at 30 ˚C for 96 h. [b]Molar ratio of cyclohexanone to 4-nitrobenzaldehyde. [c]Yield of the isolated product after silica gel chromatography. [d]Determined by chiral HPLC analysis. [e]e.e. value of the *syn*-isomer, determined by chiral HPLC using a chiralpak AD-H column.

Supplementary Table S6. Influence of temperature on the SGP-catalysed Mannich reaction[a].

Entry	T (˚C)	Yield (%)[b]	dr (*syn*:*anti*)[c]	e.e. (*syn*) (%)[d]
1	15	65	77:23	74
2	20	67	83:17	79
3	25	62	86:14	82
4	30	64	88:12	83
5	35	57	80:20	81
6	40	57	71:29	75
7	45	52	69:31	75

[a]Reaction conditions: a mixture of 4-nitrobenzaldehyde (0.5 mmol), aniline (0.55 mmol), cyclohexanone (0.75 mmol), deionised water (0.10 mL), MeCN (0.9 mL) and SGP (50 mg) was stirred at 15-45 ˚C for 96 h. [b]Yield of the isolated product after silica gel chromatography. [c]Determined by chiral HPLC analysis. [d]e.e. value of the *syn*-isomer, determined by chiral HPLC using a chiralpak AD-H column.

Supplementary Table S7. Time course of the SGP-catalysed Mannich Reaction[a].

Entry	Time (h)	Yield (%)[b]	dr (*syn*:*anti*)[c]	e.e. (*syn*) (%)[d]

1	12	13	89:11	79
2	24	45	83:17	78
3	36	49	87:13	78
4	48	54	89:11	82
5	72	57	84:16	81
6	96	64	88:12	83
7	120	66	82:18	75
8	144	67	79:21	76

[a]Reaction conditions: a mixture of 4-nitrobenzaldehyde (0.5 mmol), aniline (0.55 mmol), cyclohexanone (7.5 mmol), deionised water (0.10 mL), MeCN (0.9 mL) and SGP (50 mg) was stirred at 30 ˚C for 12-144 h. [b]Yield of the isolated product after silica gel chromatography. [c]Determined by chiral HPLC analysis. [d]e.e. value of the *syn*-isomer, determined by chiral HPLC using a chiralpak AD-H column.

3. Characterization of the Mannich products

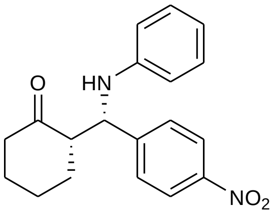

2-[(4-nitrophenyl)(phenylamino)methyl]yclohexanone (*syn*)[1] (4a)

^{1}H NMR (300 MHz, $CDCl_3$): δ = 8.16-8.13 (m, 2H), 7.57-7.54 (m, 2H), 7.11-7.05 (m, 2H), 6.70-6.65 (m, 1H), 6.51-6.48 (m, 2H), 4.86-4.59 (m, 2H), 2.86-2.83 (m, 1H), 2.46-2.27 (m, 2H), 2.06-1.93 (m, 3H), 1.72-1.57 (m, 3H) ppm; ^{13}C NMR (75 MHz, $CDCl_3$): δ = 210.6, 149.6, 147.0, 146.6, 129.1, 128.6, 123.6, 118.3, 114.0, 57.1, 56.2, 42.4, 29.0, 27.0, 24.9 ppm; The enantiomeric excess was determined by HPLC (Daicel Chiralpak AD-H, hexane/isopropanol = 80:20, flow rate 1.0 mL/min, λ = 254 nm), *syn*-diastereomer: t_R = 12.550 min (minor), 15.850 min (major).

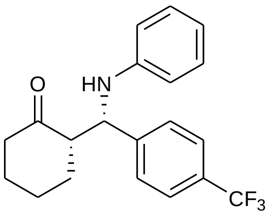

2-[(phenylamino)(4-(trifluoromethyl)phenyl)methyl]cyclohexanone (*syn*)[1] (4b)

^{1}H NMR (300 MHz, $CDCl_3$): δ = 7.56-7.47 (m, 4H), 7.10-7.06 (m, 2H), 6.69-6.65 (m, 1H), 6.53-6.50 (m, 2H), 4.83-4.58 (m, 2H), 2.81-2.31 (m, 3H), 2.04-1.92 (m, 3H), 1.76-1.61 (m, 3H) ppm; ^{13}C NMR

(75 MHz, $CDCl_3$): δ = 210.9, 146.9, 145.8, 129.1, 127.9, 125.3, 125.3, 118.0, 114.0, 57.1, 56.3, 42.4, 28.8, 27.0, 24.9 ppm; The enantiomeric excess was determined by HPLC (Daicel Chiralpak AS-H, hexane/isopropanol = 90:10, flow rate 1.0 mL/min, λ = 254 nm), *syn*-diastereomer: t_R = 10.946 min (minor), 14.012 min (major).

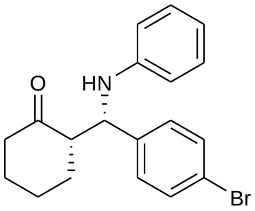

2-[(4-bromophenyl)(phenylamino)methyl]cyclohexanone (*syn*)[1] (4c)

^{1}H NMR (300 MHz, $CDCl_3$): δ = 7.41-7.39 (m, 2H), 7.25-7.22 (m, 2H), 7.10-7.05 (m, 2H), 6.68-6.63 (m, 1H), 6.52-6.49 (m, 2H), 4.71-4.55 (m, 2H), 2.76-2.39 (m, 2H), 2.32-2.24 (m,1H), 2.02-1.89 (m, 3H), 1.60-1.54 (m, 3H) ppm; ^{13}C NMR (75 MHz, $CDCl_3$): δ = 211.1, 147.1, 140.5, 131.4, 129.4, 129.0, 120.8, 117.9, 114.0, 56.9, 56.3, 42.4, 28.8, 27.0, 24.8 ppm; The enantiomeric excess was determined by HPLC (Daicel Chiralpak AD-H, hexane/isopropanol = 95:5, flow rate 1.0 mL/min, λ = 254 nm), *syn*-diastereomer: t_R = 14.856 min (minor), 18.884 min (major).

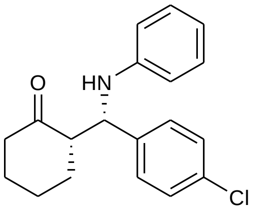

2-[(4-chlorophenyl)(phenylamino)methyl]cyclohexanone (*syn*)[1] (4d)

^{1}H NMR (300 MHz, $CDCl_3$): δ = 7.31-7.23 (m, 4H), 7.10-7.05 (m, 2H), 6.68-6.63 (m, 1H), 6.52-6.50 (m, 2H), 4.72-4.55 (m, 2H), 2.77-2.39 (m, 3H), 2.33-1.89 (m, 3H), 1.61-1.55 (m, 3H) ppm; ^{13}C NMR (75 MHz, $CDCl_3$): δ = 211.2, 147.1, 140.0, 132.6, 129.0, 129.0, 128.5, 117.9, 114.0, 56.9, 56.3, 42.4, 28.9, 27.0, 24.8 ppm; The enantiomeric excess was determined by HPLC (Daicel Chiralpak AD-H, hexane/isopropanol = 95:5, flow rate 1.0 mL/min, λ = 254 nm), *syn*-diastereomer: t_R = 13.139 min (minor), 16.600 min (major).

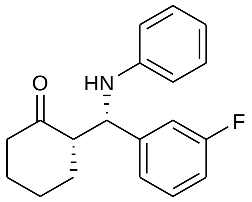

2-[(3-fluorophenyl)(phenylamino)methyl]cyclohexanone (*syn*)[1] (4e)

^{1}H NMR (300 MHz, $CDCl_3$): δ = 7.26-7.22 (m, 2H), 7.14-7.06 (m, 3H), 6.93-6.87 (m, 1H), 6.69-6.64 (m, 1H), 6.54-6.52 (m, 2H), 4.77-4.51 (m, 2H), 2.79-2.31 (m, 3H), 2.05-1.92 (m, 3H), 1.69-1.62 (m, 3H) ppm; ^{13}C NMR (75 MHz, $CDCl_3$): δ = 211.0, 161.4, 147.2, 144.6, 129.8, 129.7, 129.0, 123.1, 117.9, 114.0, 113.8, 56.9, 56.4, 42.4, 28.6, 27.0, 24.8 ppm; The enantiomeric excess was determined by HPLC (Daicel Chiralcel OJ-H, hexane/isopropanol = 85:15, flow rate 1.0 mL/min, λ = 254 nm), *syn*-diastereomer: t_R = 16.212 min (minor), 23.008 min (major).

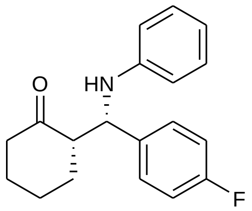

2-[(4-fluorophenyl)(phenylamino)methyl]cyclohexanone (*syn*)[2] (4f)

^{1}H NMR (300 MHz, $CDCl_3$): δ = 7.34-7.30 (m, 2H), 7.10-7.05 (m, 2H), 6.99-6.94 (m, 2H), 6.65-6.51 (m, 3H), 4.73-4.57 (m, 2H), 2.77-2.29 (m, 3H), 2.05-1.89 (m, 3H), 1.61-1.59 (m, 3H) ppm; ^{13}C NMR (75 MHz, $CDCl_3$): δ = 211.3, 160.1, 147.2, 137.0, 129.1, 117.8, 115.3, 114.0, 56.8, 56.4, 42.4, 28.9, 27.0, 24.8 ppm; The enantiomeric excess was determined by HPLC (Daicel Chiralpak AD-H, hexane/isopropanol = 90:10, flow rate 1.0 mL/min, λ = 254 nm), *syn*-diastereomer: t_R = 9.577 min (minor), 13.237 min (major).

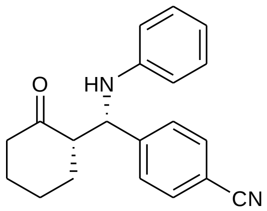

4-[(2-oxocyclohexyl)(phenylamino)methyl]benzonitrile (*syn*)[1] (4g)

H NMR (300 MHz, $CDCl_3$): δ = 7.58-7.56 (m, 2H), 7.52-7.50 (m, 2H), 7.10-7.05 (m, 2H), 6.68-6.65

(m, 1H), 6.50-6.47 (m, 2H), 4.85-4.67 (m, 2H), 2.83-2.38 (m, 3H), 2.04-1.94 (m, 3H), 1.73-1.62 (m, 3H) ppm; ^{13}C NMR (75 MHz, $CDCl_3$): δ = 211.9, 147.7, 146.7, 132.2, 129.2, 128.1, 118.8, 117.9, 113.4, 110.8, 57.8, 57.0, 42.3, 31.9, 27.8, 24.4 ppm; The enantiomeric excess was determined by HPLC (Daicel Chiralpak AS-H, hexane/isopropanol = 80:20, flow rate 0.5 mL/min, λ = 254 nm), *syn*-diastereomer: t_R = 38.179 min (minor), 95.360 min (major).

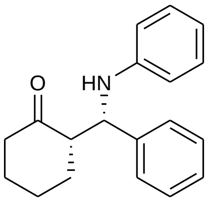

2-[phenyl(phenylamino)methyl]cyclohexanone (*syn*)[2] (4h)

^{1}H NMR (300 MHz, $CDCl_3$): δ = 7.35-7.19 (m, 5H), 7.09-7.04 (m, 2H), 6.66-6.53 (m, 3H), 4.80-4.56 (m, 2H), 2.78-2.76 (m, 1H), 2.43-2.23 (m, 2H), 2.03-1.88 (m, 3H), 1.67-1.57 (m, 3H) ppm; ^{13}C NMR (75 MHz, $CDCl_3$): δ = 211.3, 147.4, 141.5, 129.0, 128.3, 127.5, 117.6, 114.0, 57.2, 56.6, 42.4, 28.6, 27.0, 24.8 ppm; The enantiomeric excess was determined by HPLC (Daicel Chiralpak AD-H, hexane/isopropanol = 90:10, flow rate 1.0 mL/min, λ = 254 nm), *syn*-diastereomer: t_R = 8.265 min (minor), 10.080 min (major).

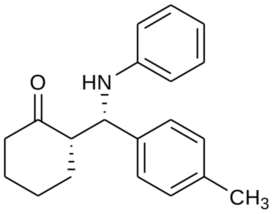

2-[4-methyl-phenyl(phenylamino)methyl]cyclohexanone (*syn*)[2] (4i)

^{1}H NMR (300 MHz, $CDCl_3$): δ = 7.24-7.21 (m, 2H), 7.15-7.04 (m, 4H), 6.69-6.61 (m, 1H), 6.56-6.53 (m, 2H), 4.76-4.51 (m, 2H), 2.78-2.75 (m, 1H), 2.42-2.39 (m, 2H), 2.29 (s, 3H), 2.04-1.89 (m, 3H), 1.68-1.58 (m, 3H) ppm; ^{13}C NMR (75 MHz, $CDCl_3$): δ = 211.5, 147.5, 138.4, 136.5, 129.0, 129.0, 127.4, 117.5, 114.0, 56.9, 56.6, 42.4, 28.7, 27.0, 24.8, 21.0 ppm; The enantiomeric excess was determined by HPLC (Daicel Chiralpak AD-H, hexane/isopropanol = 95:5, flow rate 1.0 mL/min, λ = 254 nm), *syn*-diastereomer: t_R = 12.603 min (minor), 9.640 min (major).

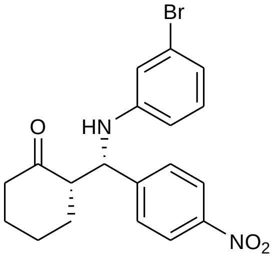

2-[(3-bromophenylamino)-(4-nitrophenyl)methyl]cyclohexanone (*syn*)[3] (4j)

^{1}H NMR (300 MHz, $CDCl_3$): δ = 8.18-8.15 (m, 2H), 7.55-7.52 (m, 2H), 6.95-6.90 (m, 1H), 6.80-6.77 (m, 1H), 6.66-6.65 (m, 1H), 6.41-6.37 (m, 1H), 4.83-4.71 (m, 2H), 2.88-2.82 (m, 1H), 2.47-2.28 (m, 2H), 2.06-1.93 (m, 2H), 1.66-1.55 (m, 4H) ppm; ^{13}C NMR (75 MHz, $CDCl_3$): δ = 210.5, 148.7, 148.3, 147.2, 130.4, 128.5, 123.7, 121.1, 116.7, 112.4, 57.0, 42.3, 32.0, 29.0, 26.9, 24.8 ppm; The enantiomeric excess was determined by HPLC (Daicel Chiralpak AD-H, hexane/isopropanol = 85:15, flow rate 0.5 mL/min, λ = 254 nm), *syn*-diastereomer: t_R = 29.970 min (minor), 41.740 min (major).

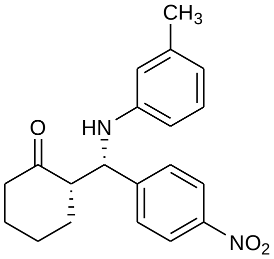

2-[(3-Methylphenylamino)-(4-nitrophenyl)methyl]cyclohexanone (*syn*)[3] (4k)

^{1}H NMR (300 MHz, $CDCl_3$): δ = 8.16-8.13 (m, 2H), 7.56-7.54 (m, 2H), 6.98-6.93 (m, 1H), 6.52-6.49 (m, 1H), 6.36 (s, 1H), 6.29-6.26 (m, 1H), 4.86-4.52 (m, 2H), 2.87-2.81 (m, 1H), 2.46-2.31 (m, 2H), 2.19 (s, 3H), 2.06-1.59 (m, 6H) ppm; ^{13}C NMR (75 MHz, $CDCl_3$): δ = 210.6, 149.7, 138.9, 128.5, 123.6, 119.3, 114.9, 110.9, 57.1, 56.2, 42.4, 29.0, 27.0, 24.9, 21.5 ppm; The enantiomeric excess was determined by HPLC (Daicel Chiralpak AD-H, hexane/isopropanol = 85:15, flow rate 1 mL/min, λ = 254 nm), *syn*-diastereomer: t_R = 16.721 min (minor), 19.124 min (major).

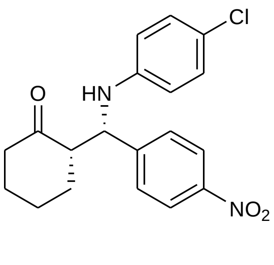

2-[(4-Chlorophenylamino)-(4-nitrophenyl)methyl]cyclohexanone (*syn*)[3] (4l)

^{1}H NMR (300 MHz, $CDCl_3$): δ = 8.16-8.14 (m, 2H), 7.52-7.51 (m, 2H), 7.03-7.00 (m, 2H), 6.43-6.40 (m, 2H), 4.81-4.66 (m, 2H), 2.87-2.83 (m, 1H), 2.47-2.35 (m, 2H), 1.75-1.61 (m, 6H) ppm; ^{13}C NMR (75 MHz, $CDCl_3$): δ = 210.6, 149.1, 149.0, 147.1, 128.9, 128.5, 123.6, 123.4, 114.6, 57.3, 56.7, 42.4, 27.8, 24.7 ppm; The enantiomeric excess was determined by HPLC (Daicel Chiralpak AD-H, hexane/isopropanol = 85:15, flow rate 1 mL/min, λ = 254 nm), *syn*-diastereomer: t_R = 37.259 min (minor), 40.626 min (major).

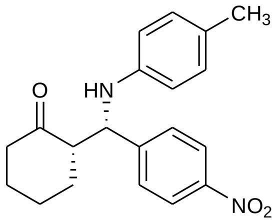

2-[(4-Methylphenylamino)-(4-nitrophenyl)methyl]cyclohexanone (*syn*)[3] (4m)

^{1}H NMR (300 MHz, $CDCl_3$): δ = 8.14-8.11 (m, 2H), 7.55-7.52 (m, 2H), 6.90-6.87 (m, 2H), 6.43-6.40 (m, 2H), 4.83-4.42 (m, 2H), 2.85-2.81 (m, 1H), 2.46-2.29 (m, 2H), 2.17 (s, 3H), 2.05-1.92 (m, 3H), 1.68-1.56 (m, 3H) ppm; ^{13}C NMR (75 MHz, $CDCl_3$): δ = 210.7, 149.8, 147.0, 129.6, 128.6, 127.6, 123.6, 113.6, 57.4, 57.0, 42.4, 29.0, 27.1, 24.9, 20.3 ppm; The enantiomeric excess was determined by HPLC (Daicel Chiralpak AD-H, hexane/isopropanol = 80:20, flow rate 1.0 mL/min, λ = 254 nm), *syn*-diastereomer: t_R = 20.458 min (minor), 21.325 min (major).

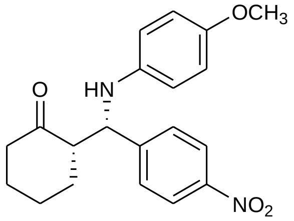

2-[(4-methoxy-phenylamino)-(4-nitro-phenyl)-methyl]-cyclohexanone (*syn*)[1] (4n)

^{1}H NMR (300 MHz, $CDCl_3$): δ = 8.15-8.12 (m, 2H), 7.55-7.52 (m, 2H), 6.68-6.65 (m, 2H), 6.47-6.45 (m, 2H), 4.80-4.64 (m, 1H), 4.29 (br, 1H), 3.67 (s, 3H), 2.82-2.32 (m, 3H), 2.04-1.92 (m, 3H), 1.72-1.61 (m, 3H) ppm; ^{13}C NMR (75 MHz, $CDCl_3$): δ = 210.8, 152.6, 149.9, 147.0, 140.7, 128.5, 123.6, 115.6, 114.6, 58.0, 56.3, 55.6, 42.4, 31.8, 27.1, 24.9 ppm; The enantiomeric excess was determined by HPLC (Daicel Chiralpak AD-H, hexane/isopropanol = 75:25, flow rate 0.5 mL/min, λ

= 254 nm), *syn*-diastereomer: t_R = 38.254 min (minor), 39.339 min (major).

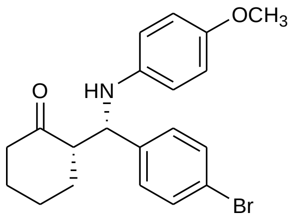

2-[(4-methoxyphenylamino)-(4-bromophenyl)methyl]cyclohexanone (*syn*)[4] (4o)

^{1}H NMR (300 MHz, $CDCl_3$): δ = 7.42-7.38 (m, 2H), 7.25-7.21 (m, 2H), 6.68-6.64 (m, 2H), 6.49-6.44 (m, 2H), 4.65-4.49 (m, 1H), 4.33 (br, 1H), 3.67 (s, 3H), 2.75-2.70 (m, 1H), 2.44-2.28 (m, 2H), 2.00-1.81 (m, 3H), 1.67-1.56 (m, 3H) ppm; ^{13}C NMR (75 MHz, $CDCl_3$): δ = 211.3, 152.4, 141.0, 140.8, 131.4, 129.4, 120.7, 115.6, 114.7, 57.8, 56.4, 55.6, 42.4, 28.6, 27.1, 24.9 ppm; The enantiomeric excess was determined by HPLC (Daicel Chiralpak AS-H, hexane/isopropanol = 80:20, flow rate 0.5 mL/min, λ = 254 nm), *syn*-diastereomer: t_R = 26.413 min (minor), 23.152min (major).

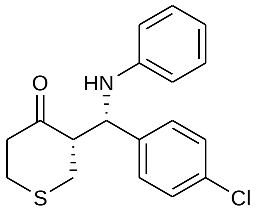

3-[(4-chlorophenyl)(phenylamino)methyl]dihydro-2H-thiopyran-4(3H)-one (*syn*)[1] (4p)

^{1}H NMR (300 MHz, $CDCl_3$): δ = 7.27-7.07 (m, 6H), 6.71-6.66 (m, 1H), 6.57-6.54 (m, 2H), 4.97-4.95 (m, 1H), 4.44 (s, 1H), 3.12-3.08 (m, 3H), 3.01-2.95 (m, 2H), 2.73-2.67 (m, 2H) ppm; ^{13}C NMR (75 MHz, $CDCl_3$): δ = 208.5, 146.6, 139.2, 133.2, 129.2, 128.9, 128.6, 118.4, 114.1, 58.4, 57.2, 44.2, 31.7, 30.3 ppm; The enantiomeric excess was determined by HPLC (Daicel Chiralcel OD-H, hexane/isopropanol = 95:5, flow rate 1.0 mL/min, λ = 254 nm), *syn*-diastereomer: t_R = 51.633 min (minor), 29.417 min (major).

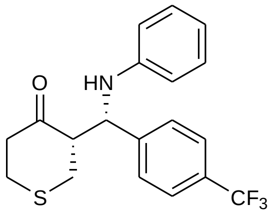

3-[(phenylamino)(4-(trifluoromethyl)phenyl)methyl]dihydro-2H-thiopyran-4(3H)-one (*syn*)[1] (4q)

^{1}H NMR (300 MHz, $CDCl_3$): δ = 7.57-7.45 (m, 4H), 7.13-7.08 (m, 2H), 6.72-6.67 (m, 1H), 6.57-6.54 (m, 2H), 5.04-5.02 (m, 1H), 4.49 (s, 1H), 3.19-3.13 (m, 1H), 3.02-2.92 (m, 4H), 2.75-2.69 (m, 2H) ppm; ^{13}C NMR (75 MHz, $CDCl_3$): δ = 208.2, 146.4, 144.9, 129.9, 129.2, 127.6, 125.7, 125.7, 118.5, 114.1, 58.4, 57.4, 44.3, 31.6, 30.3 ppm; The enantiomeric excess was determined by HPLC (Daicel Chiralpak AS-H, hexane/isopropanol = 90:10, flow rate 1.0 mL/min, λ = 254 nm), *syn*-diastereomer: t_R = 11.263 min (minor), 24.661 min (major).

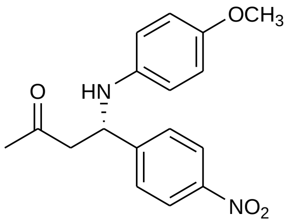

4-(4-methoxyphenylamino)-4-(4-nitrophenyl)butan-2-one[5] (4r)

^{1}H NMR (300 MHz, $CDCl_3$): δ = 8.19-8.16 (m, 2H), 7.57-7.54 (m, 2H), 6.70-6.67 (m, 2H), 6.47-6.44 (m, 2H), 4.88-4.84 (m, 1H), 4.16-4 .09 (m, 1H), 3.69 (s, 3H), 2.96-2.94 (m, 2H), 2.15 (s, 3H) ppm; ^{13}C NMR (75 MHz, $CDCl_3$): δ = 206.0, 152.8, 150.6, 147.2, 140.1, 127.4, 124.0, 115.4, 114.8, 55.6, 54.6, 50.6, 30.6 ppm; The enantiomeric excess was determined by HPLC (Daicel Chiralpak AD-H, hexane/isopropanol = 50:50, flow rate 0.5 mL/min, λ = 280 nm), t_R(S) = 19.668 min, t_R(R) = 15.923 min.

4. ^{1}H NMR, ^{13}C NMR and HPLC spectra of the Mannich products 4a-4r

^{1}H NMR Spectrum ($CDCl_3$) of **4a**

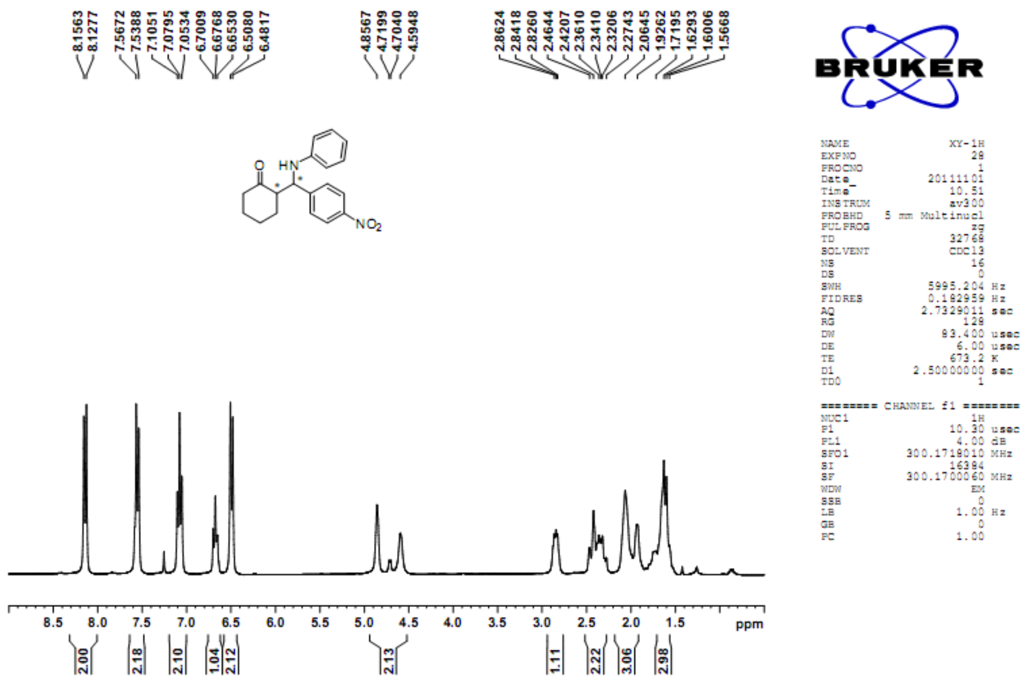

^{13}C NMR Spectrum ($CDCl_3$) of **4a**

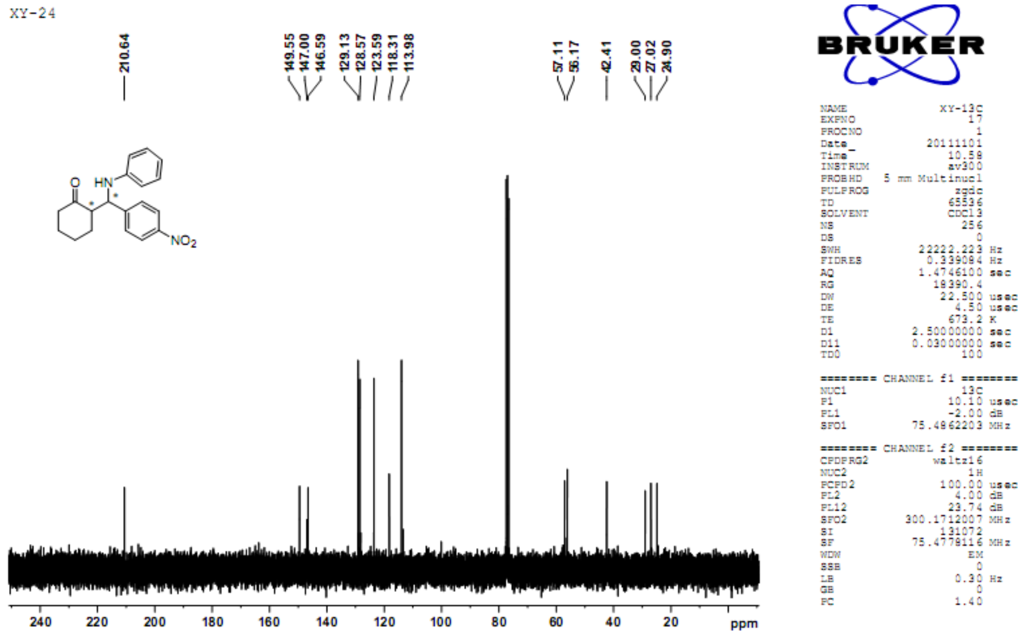

^{1}H NMR Spectrum ($CDCl_3$) of **4b**

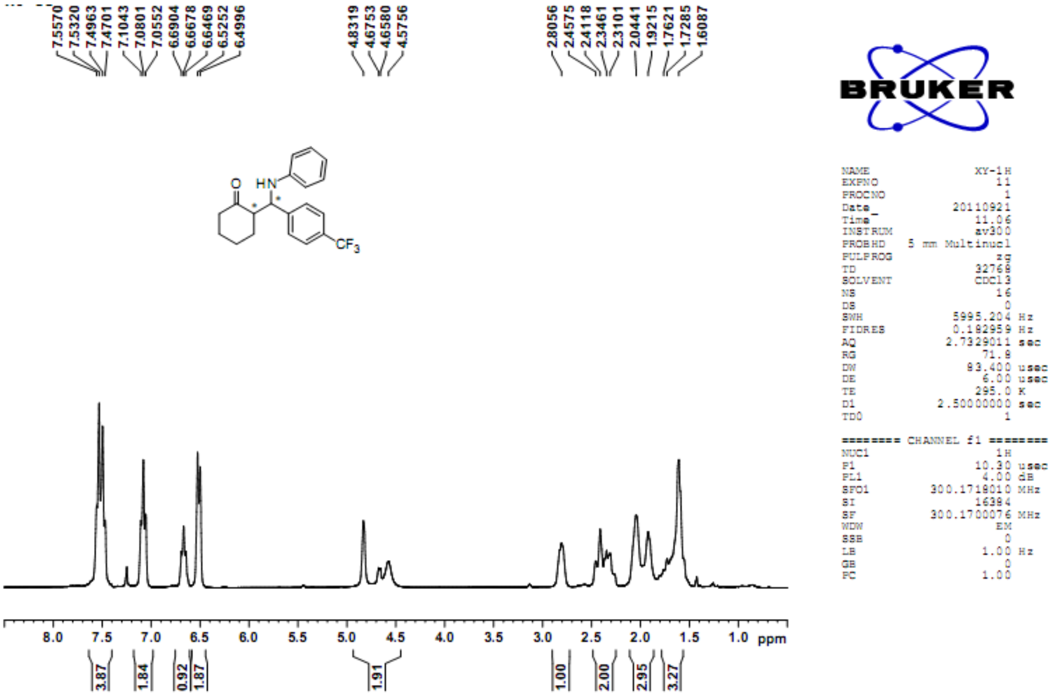

^{13}C NMR Spectrum (CDCl$_3$) of **4b**

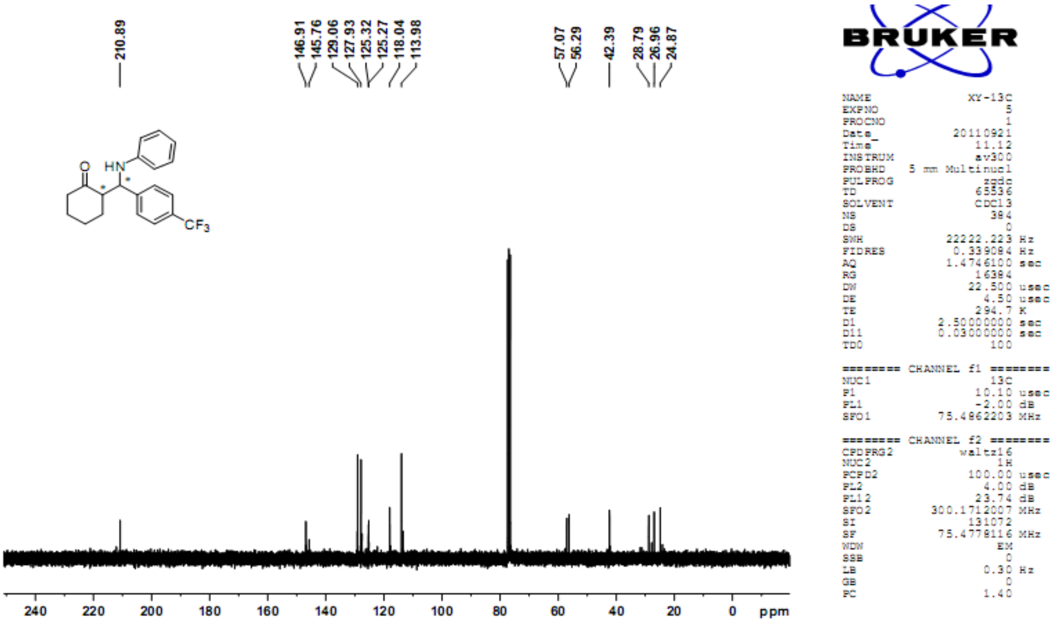

^{1}H NMR Spectrum (CDCl$_3$) of **4c**

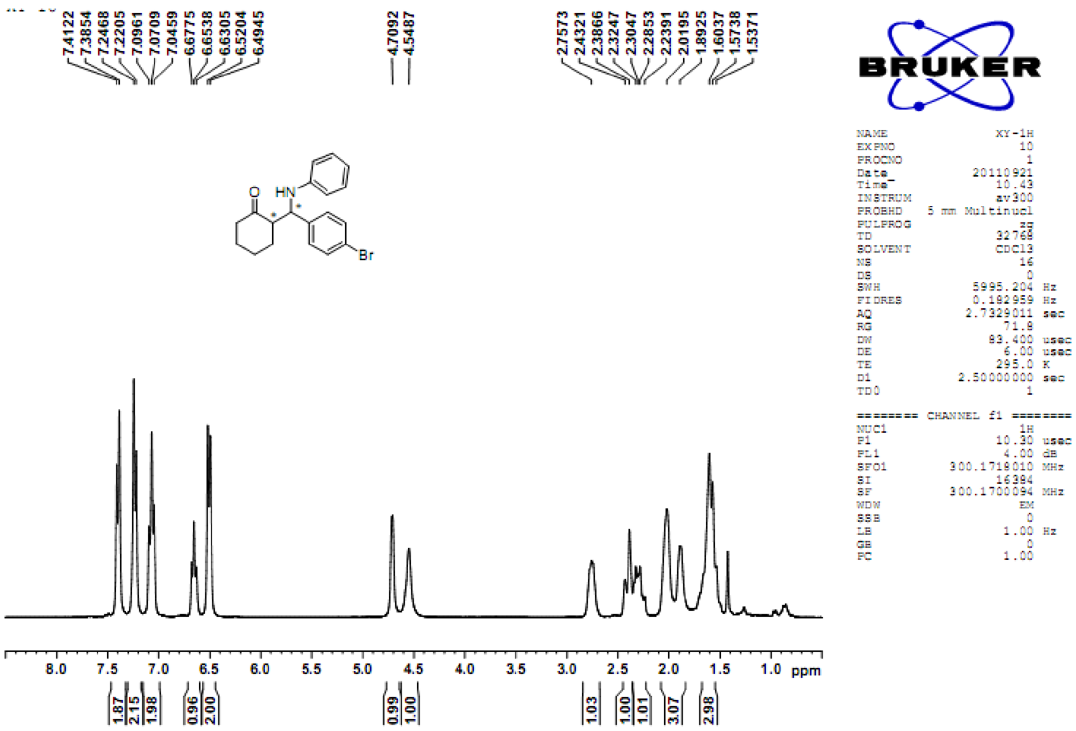

^{13}C NMR Spectrum ($CDCl_3$) of **4c**

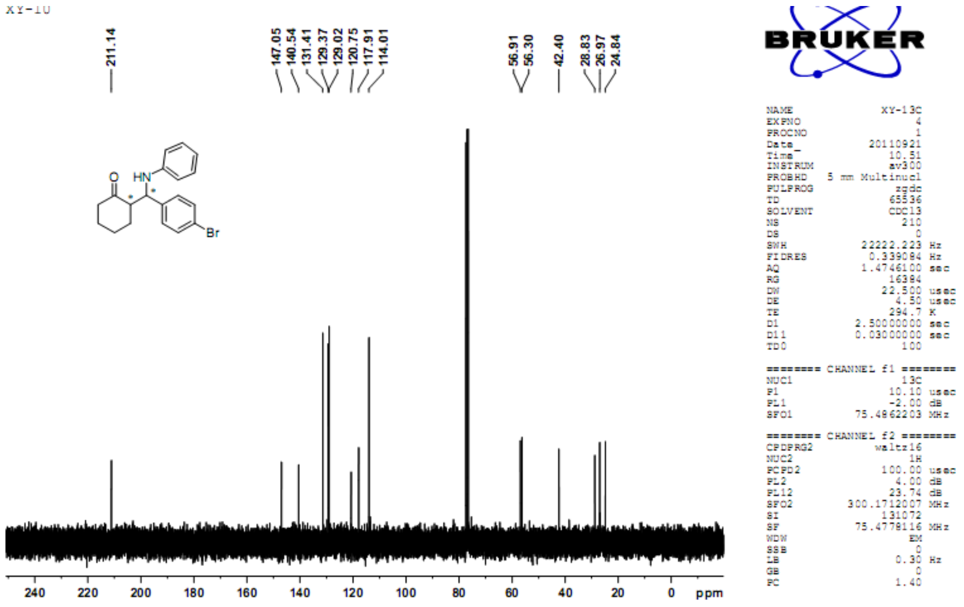

^{1}H NMR Spectrum ($CDCl_3$) of **4d**

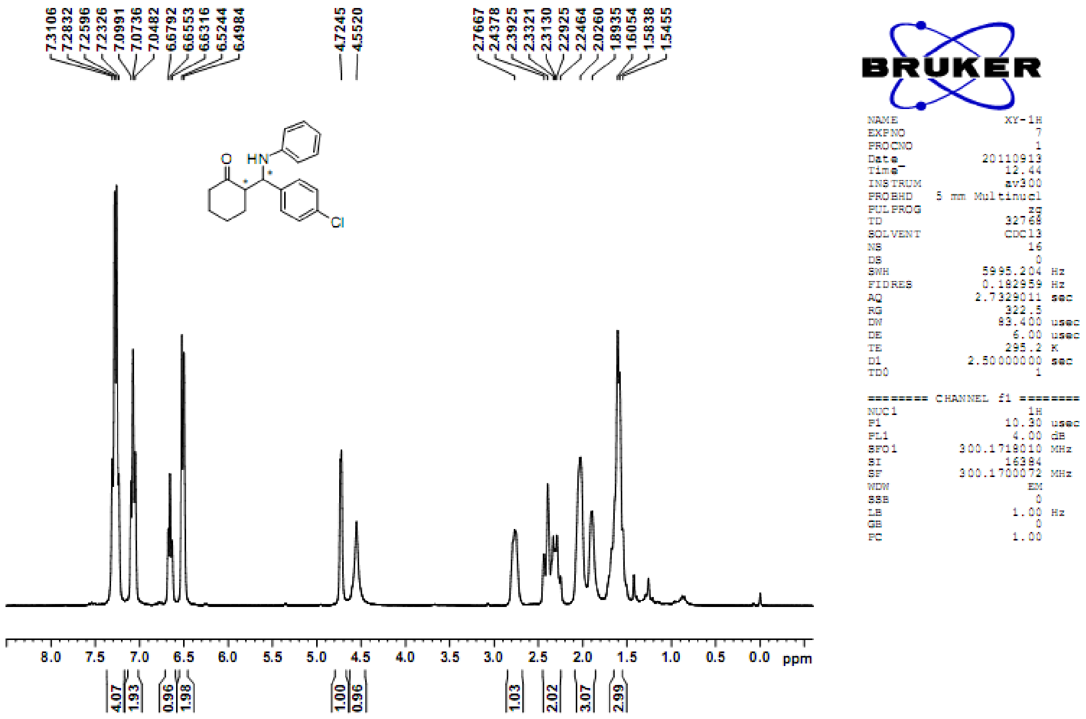

^{13}C NMR Spectrum ($CDCl_3$) of **4d**

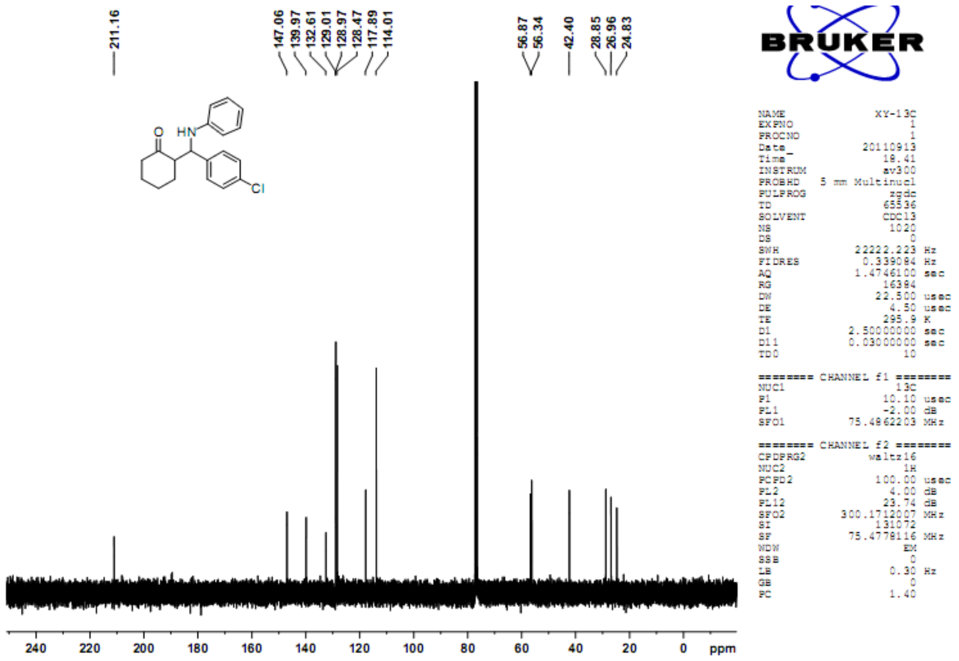

^{1}H NMR Spectrum ($CDCl_3$) of **4e**

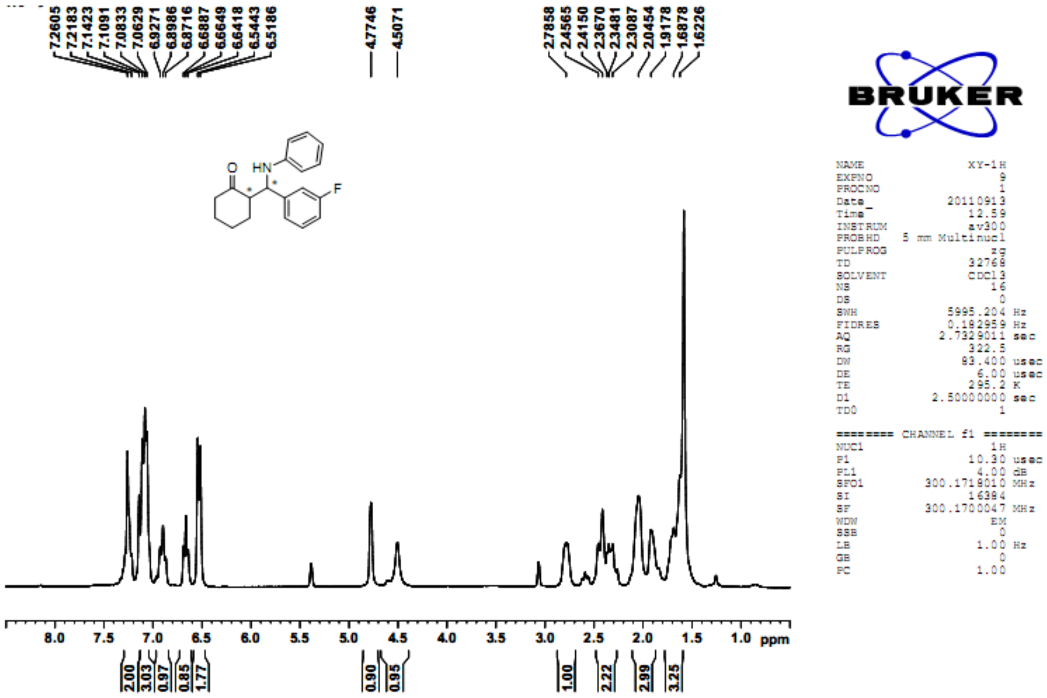

^{13}C NMR Spectrum ($CDCl_3$) of **4e**

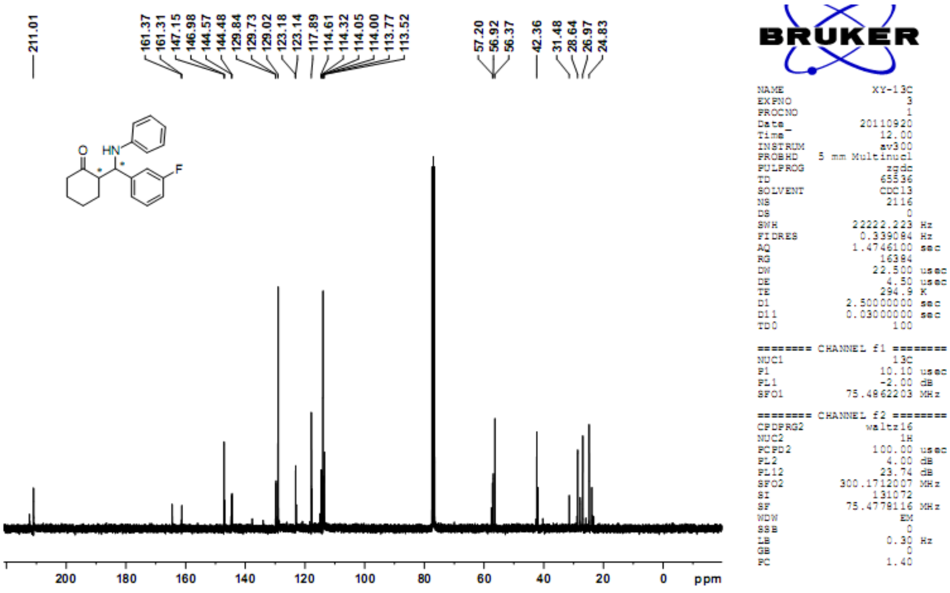

^{1}H NMR Spectrum ($CDCl_3$) of **4f**

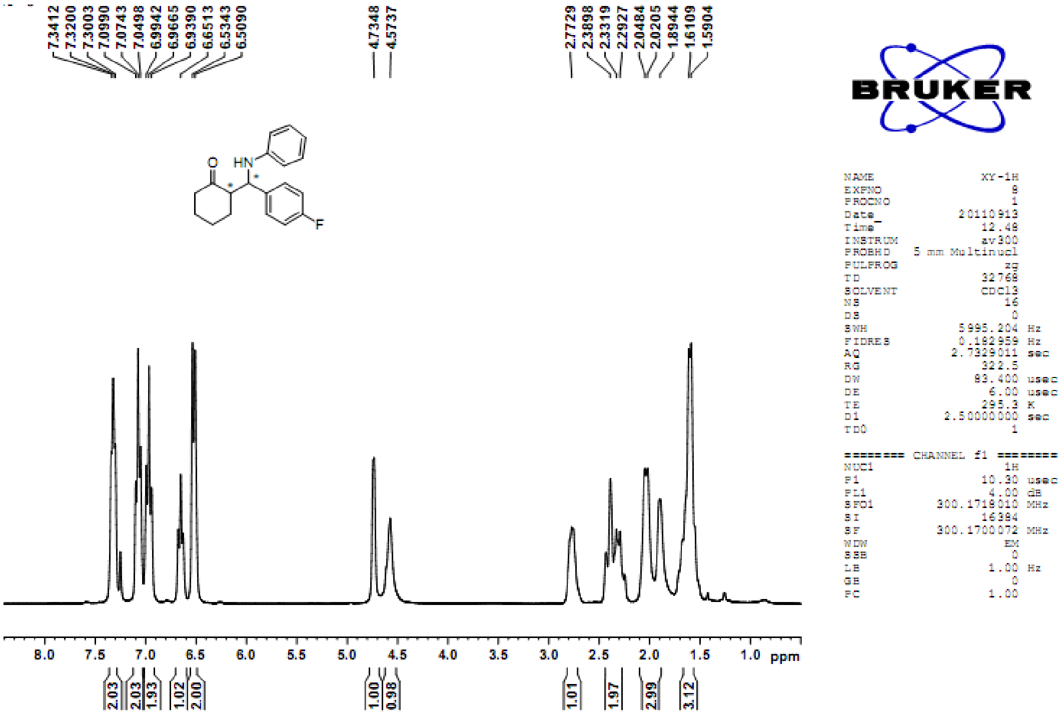

^{13}C NMR Spectrum ($CDCl_3$) of **4f**

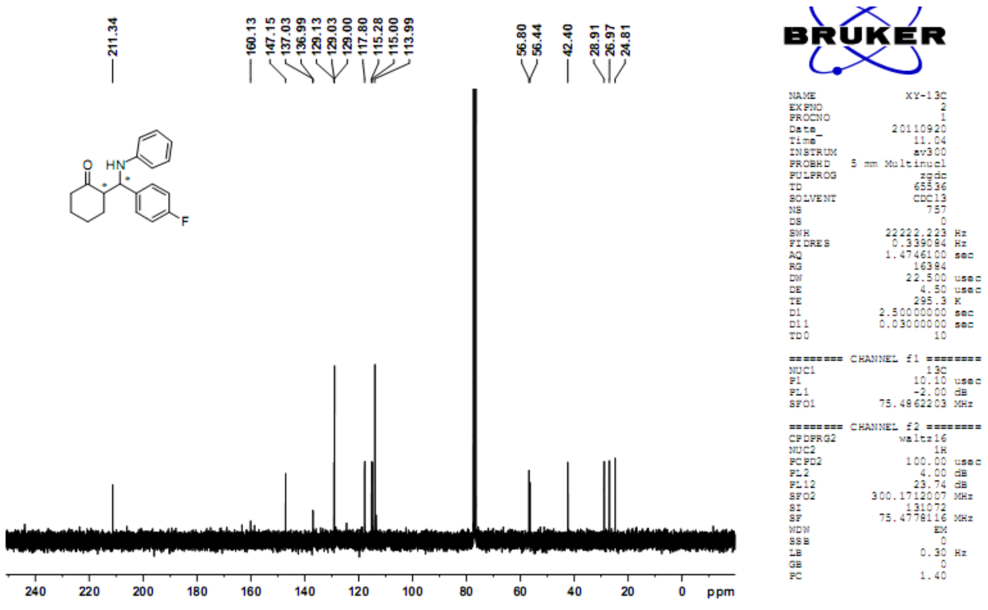

^{1}H NMR Spectrum ($CDCl_3$) of **4g**

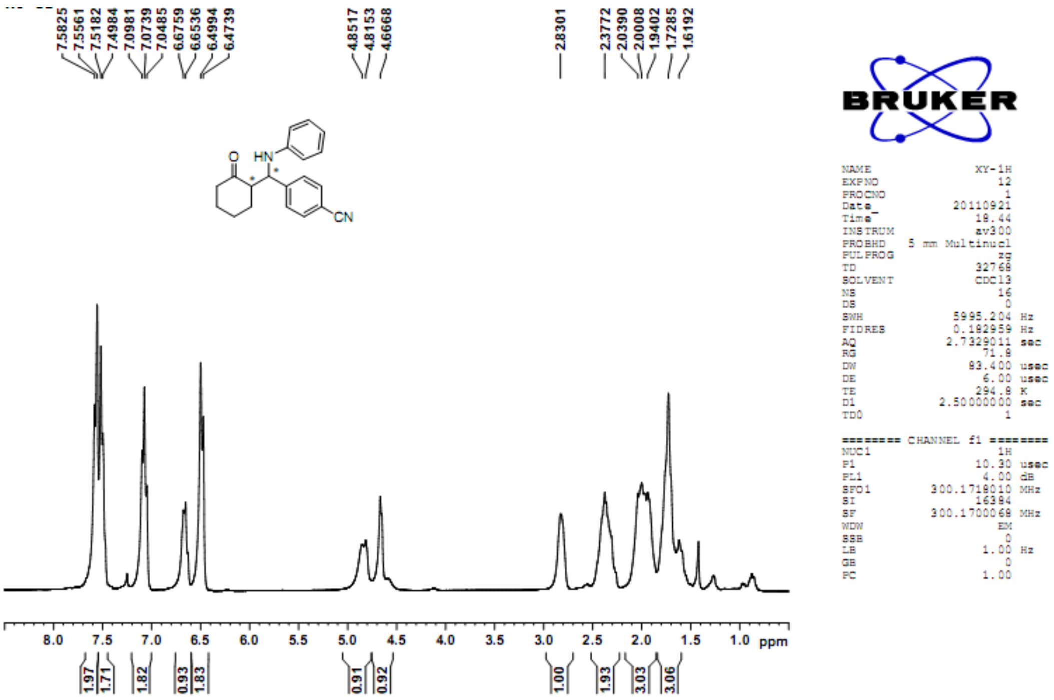

^{13}C NMR Spectrum ($CDCl_3$) of **4g**

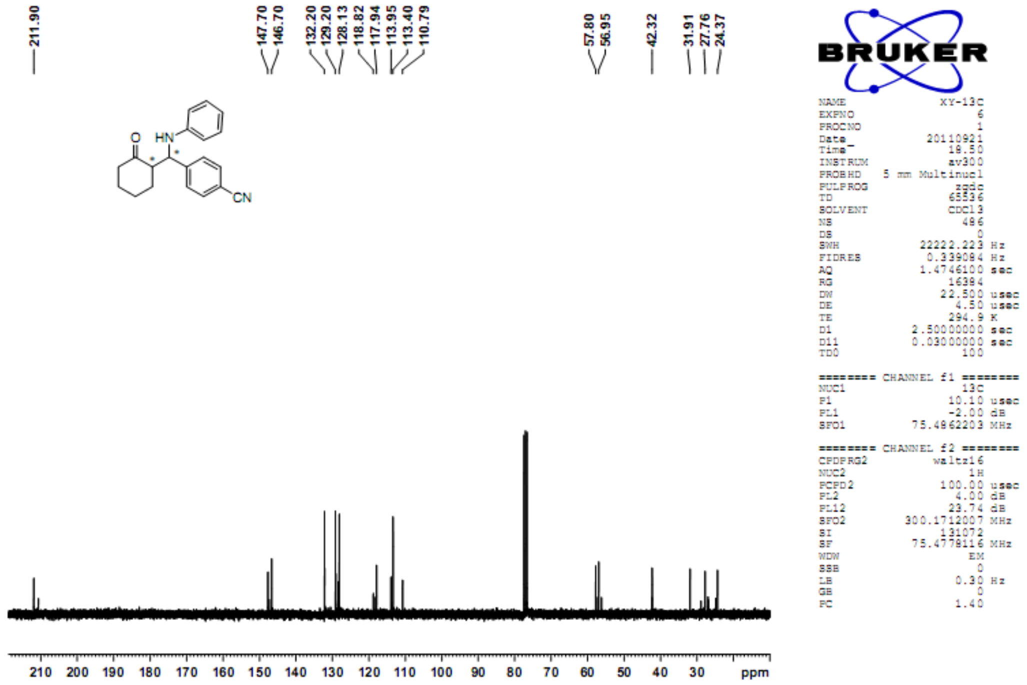

^{1}H NMR Spectrum ($CDCl_3$) of **4h**

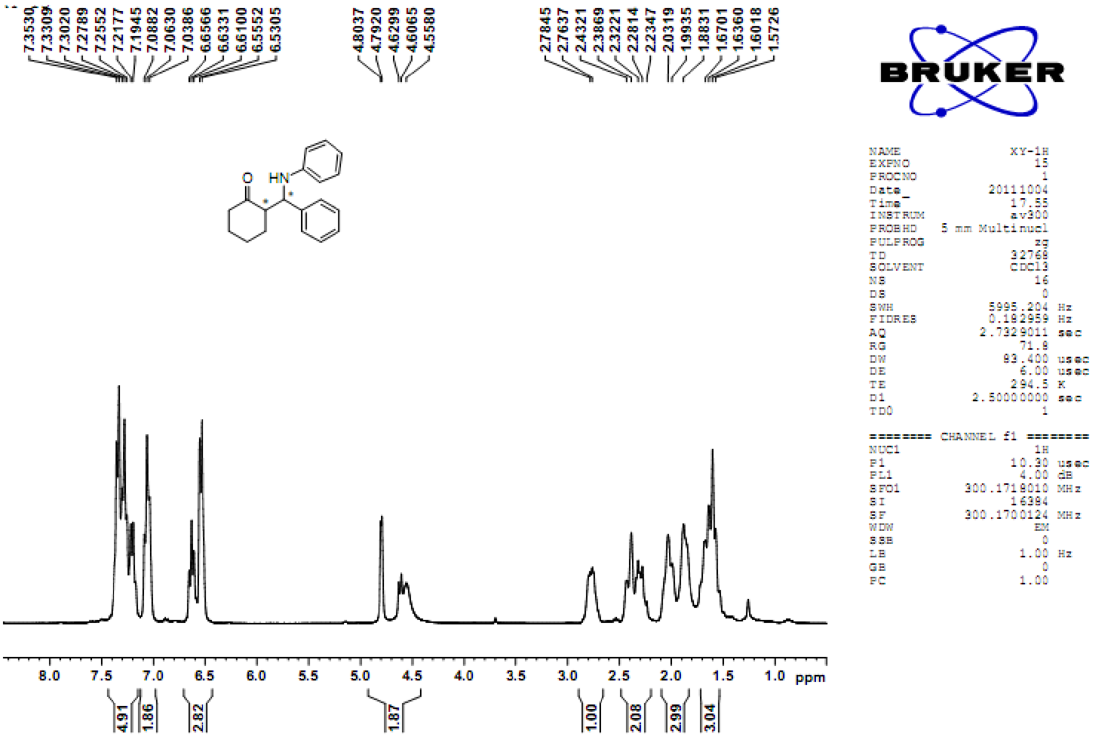

^{13}C NMR Spectrum ($CDCl_3$) of **4h**

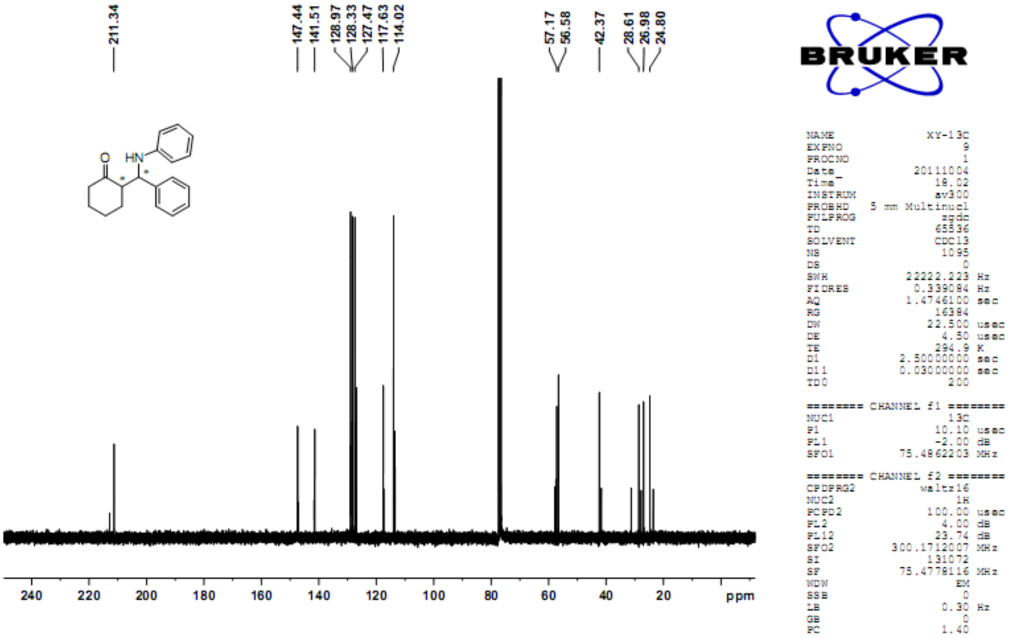

^{1}H NMR Spectrum ($CDCl_3$) of **4i**

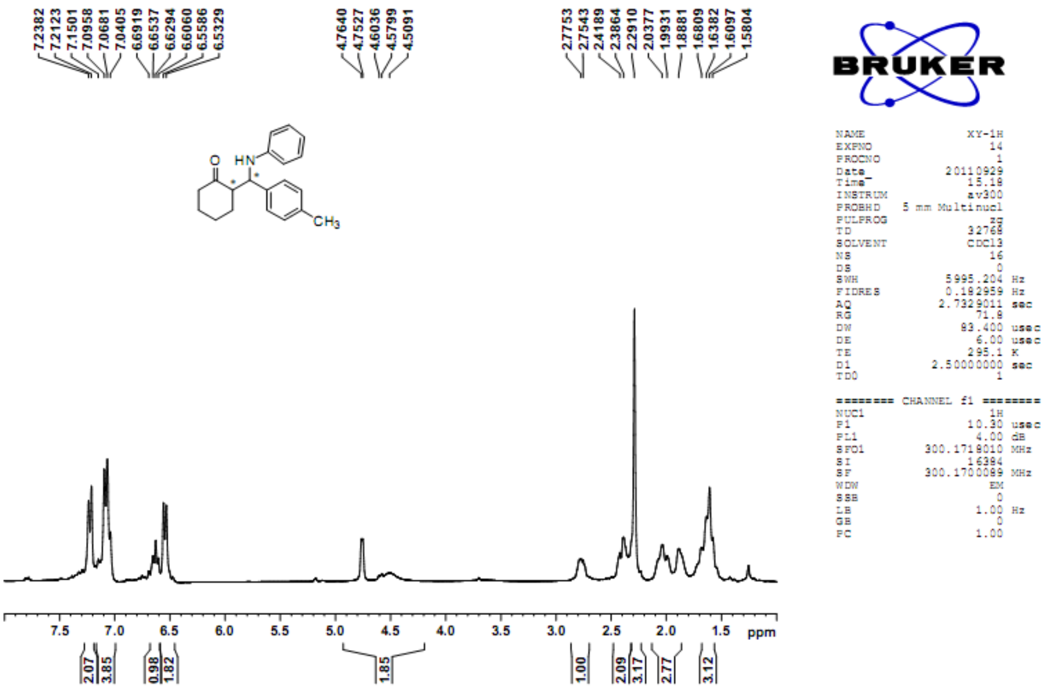

^{13}C NMR Spectrum ($CDCl_3$) of **4i**

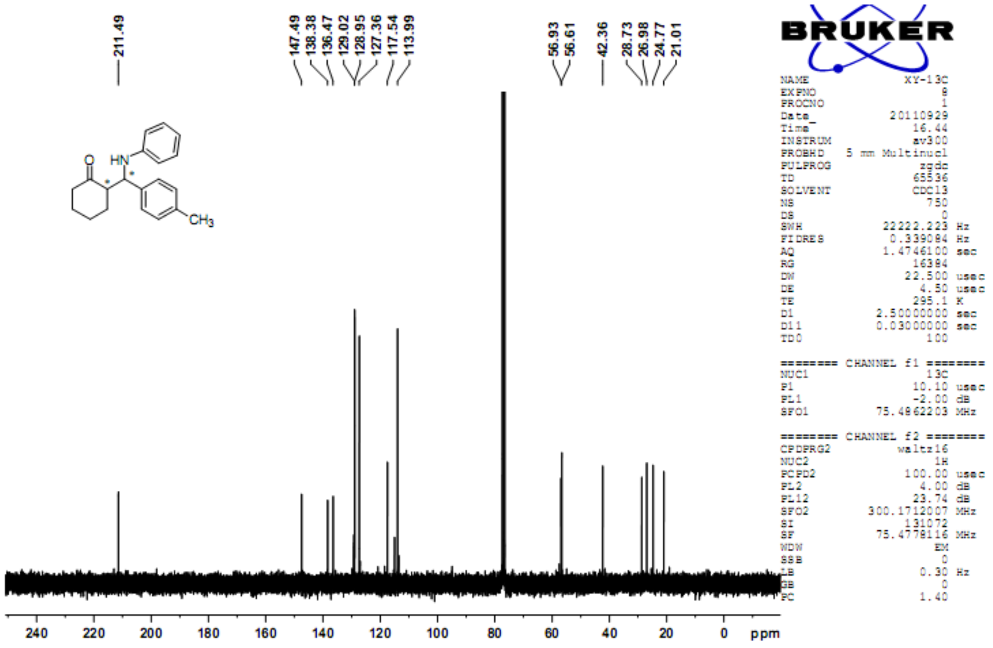

^{1}H NMR Spectrum ($CDCl_3$) of **4j**

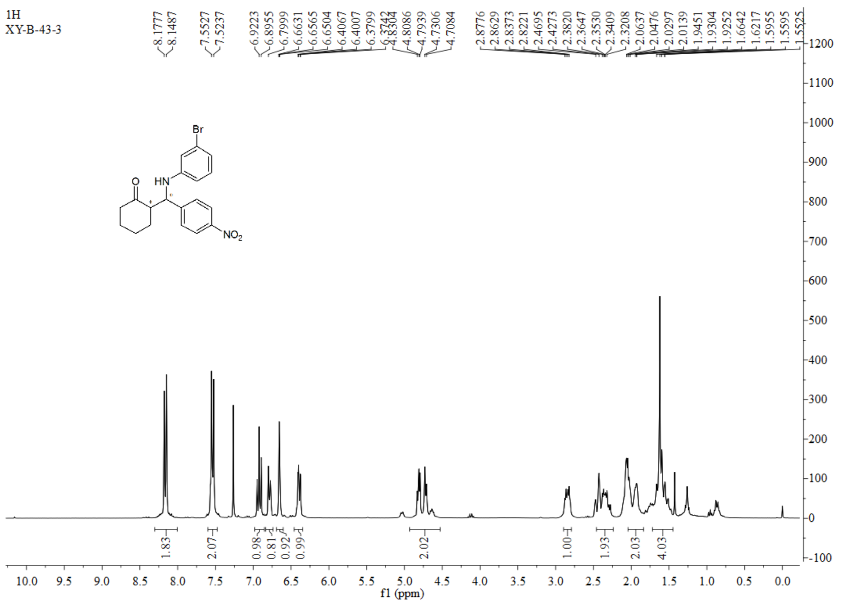

^{13}C NMR Spectrum (CDCl$_3$) of **4j**

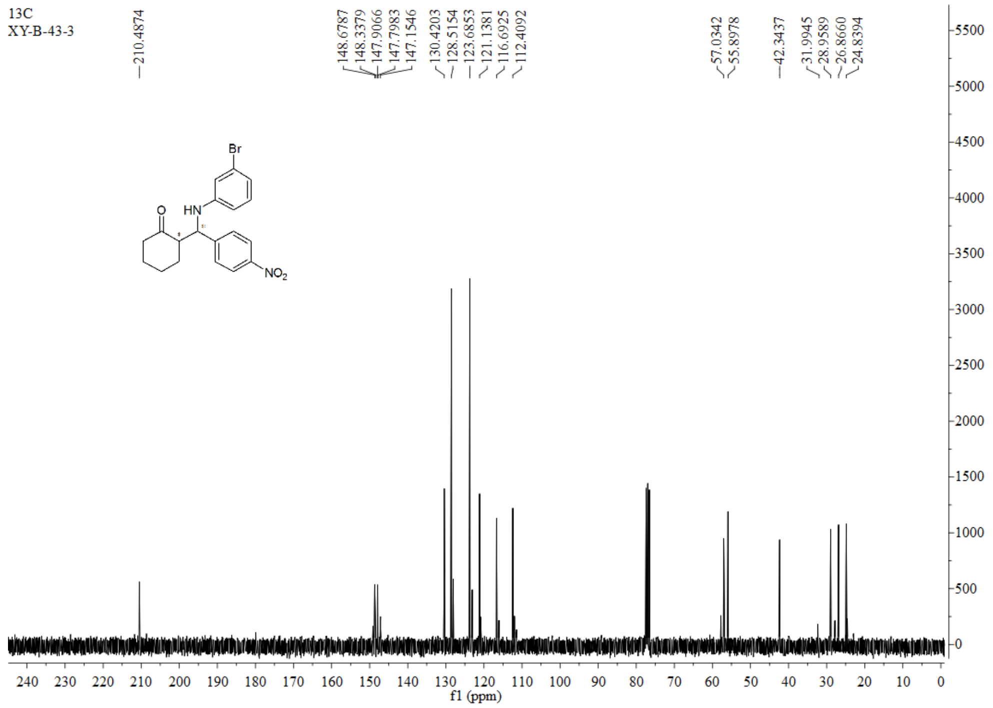

^{1}H NMR Spectrum (CDCl$_3$) of **4k**

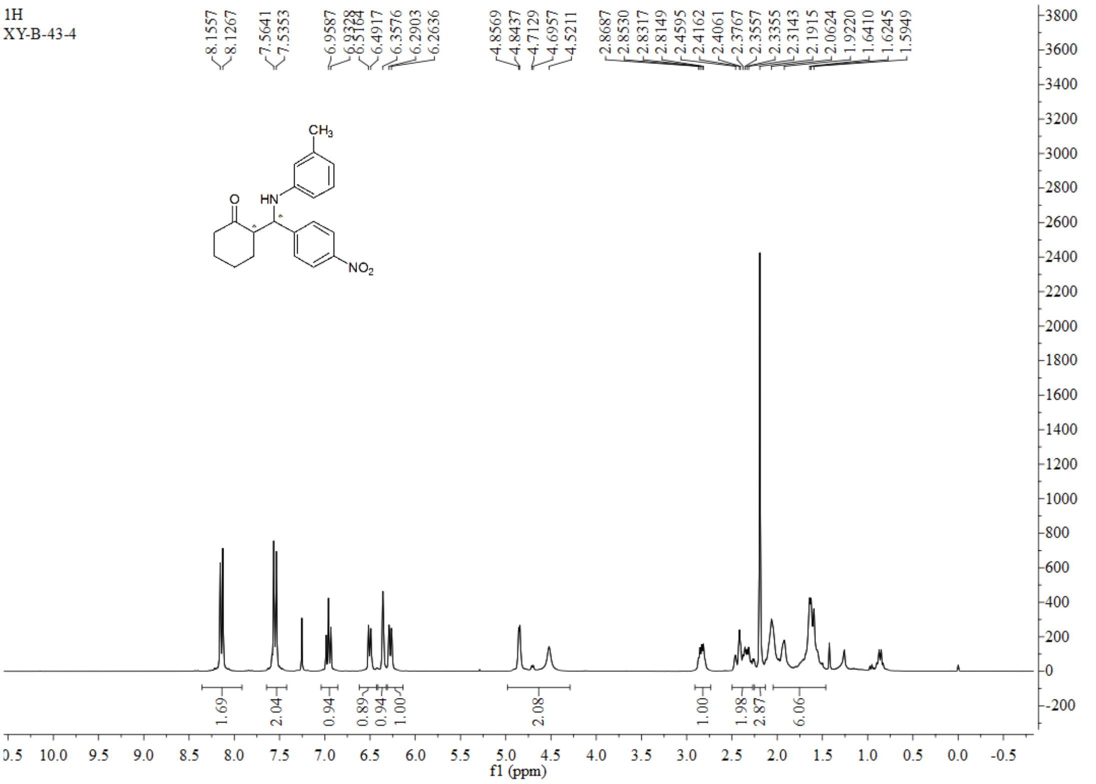

^{13}C NMR Spectrum ($CDCl_3$) of **4k**

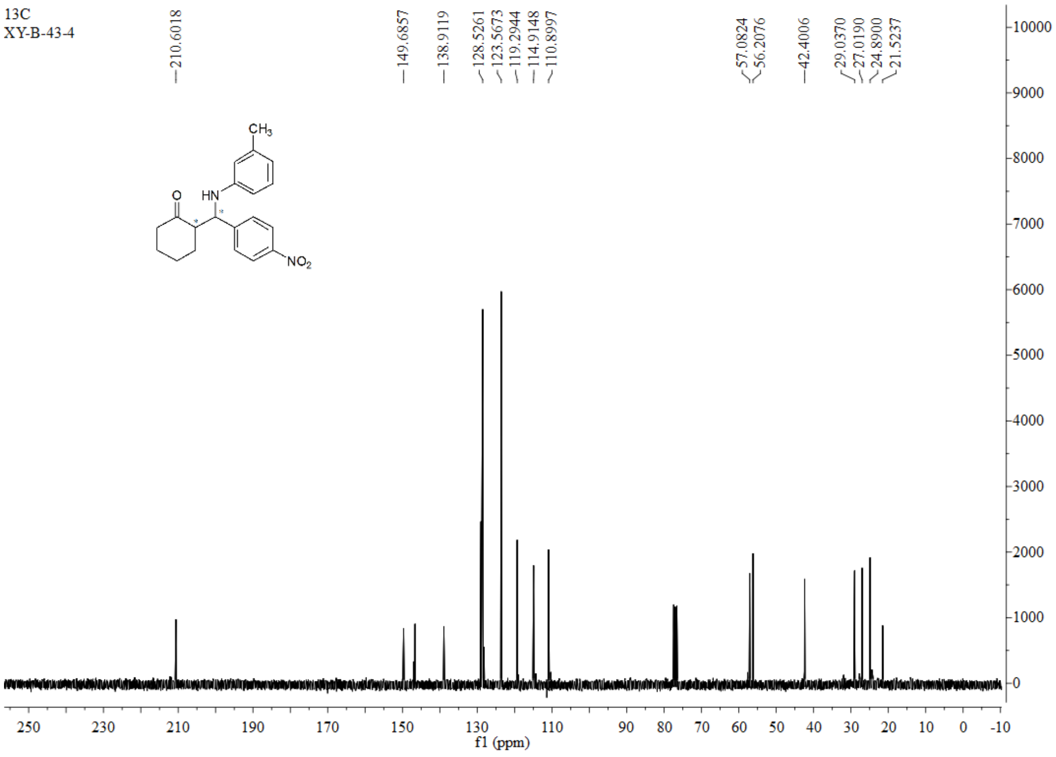

^{1}H NMR Spectrum ($CDCl_3$) of **4l**

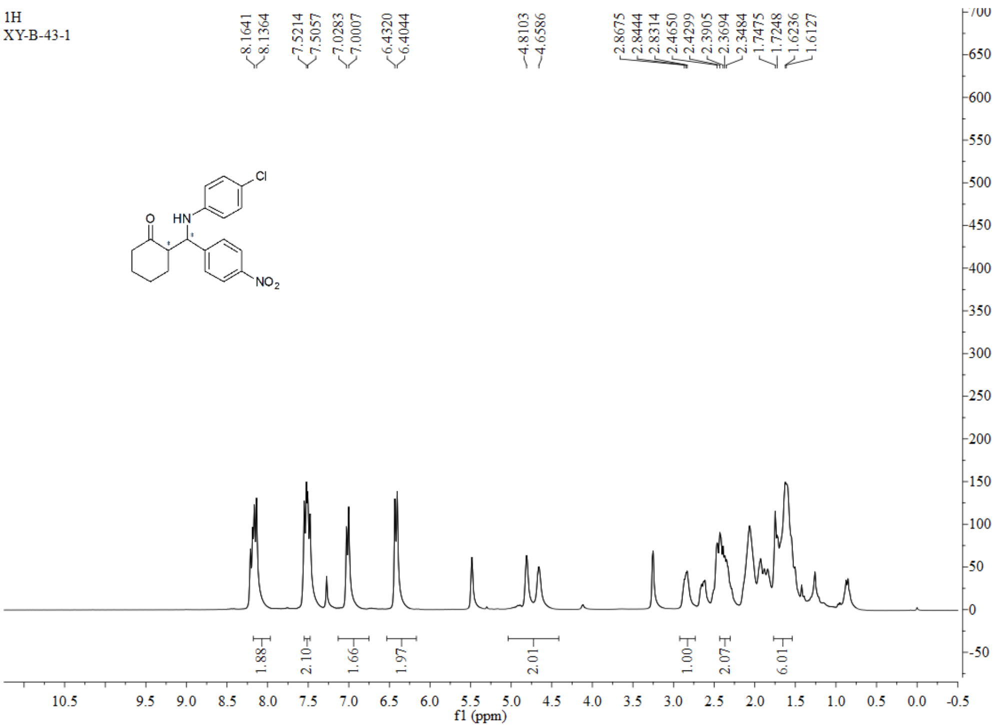

^{13}C NMR Spectrum ($CDCl_3$) of **4l**

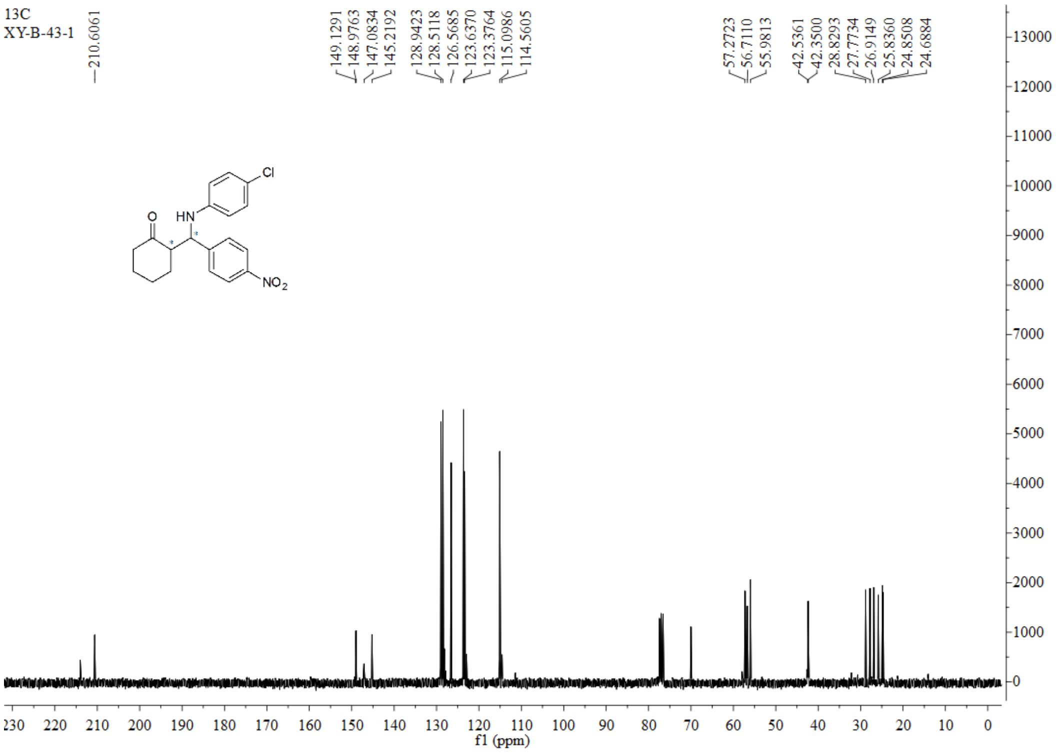

^{1}H NMR Spectrum ($CDCl_3$) of **4m**

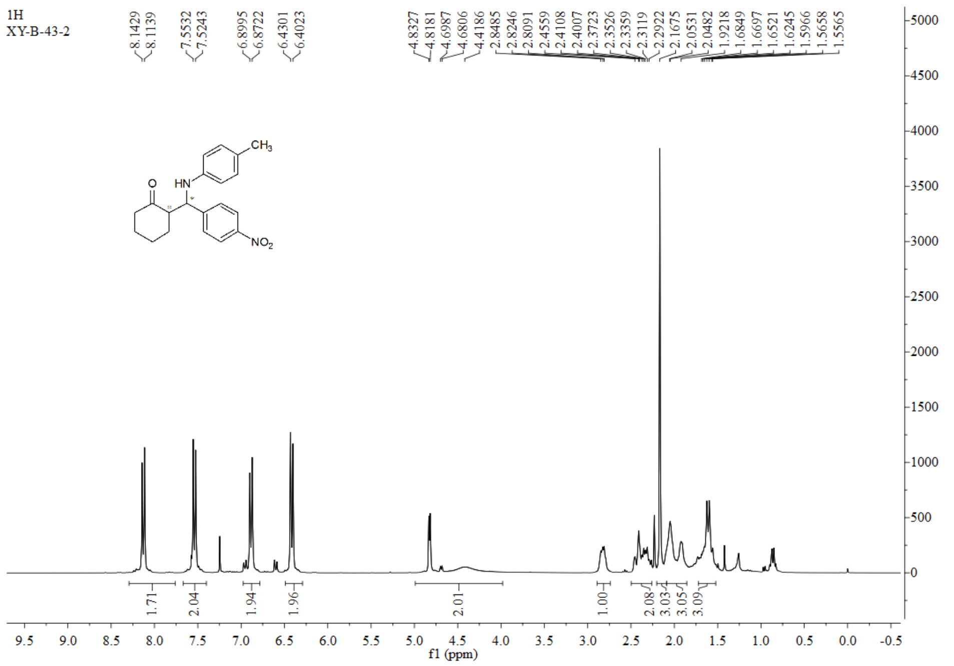

^{13}C NMR Spectrum ($CDCl_3$) of **4m**

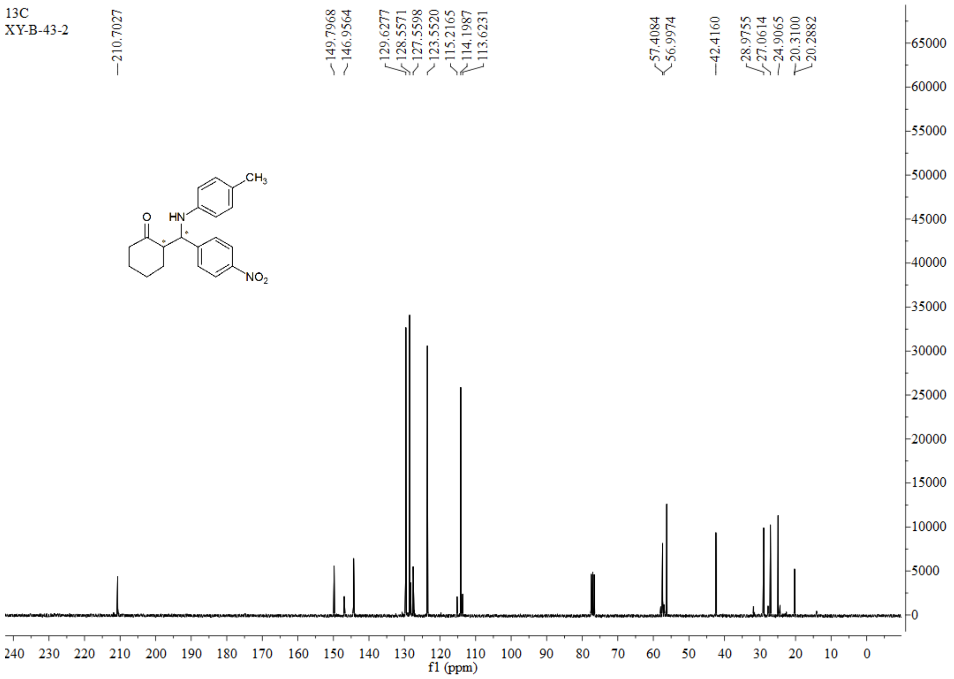

^{1}H NMR Spectrum ($CDCl_3$) of **4n**

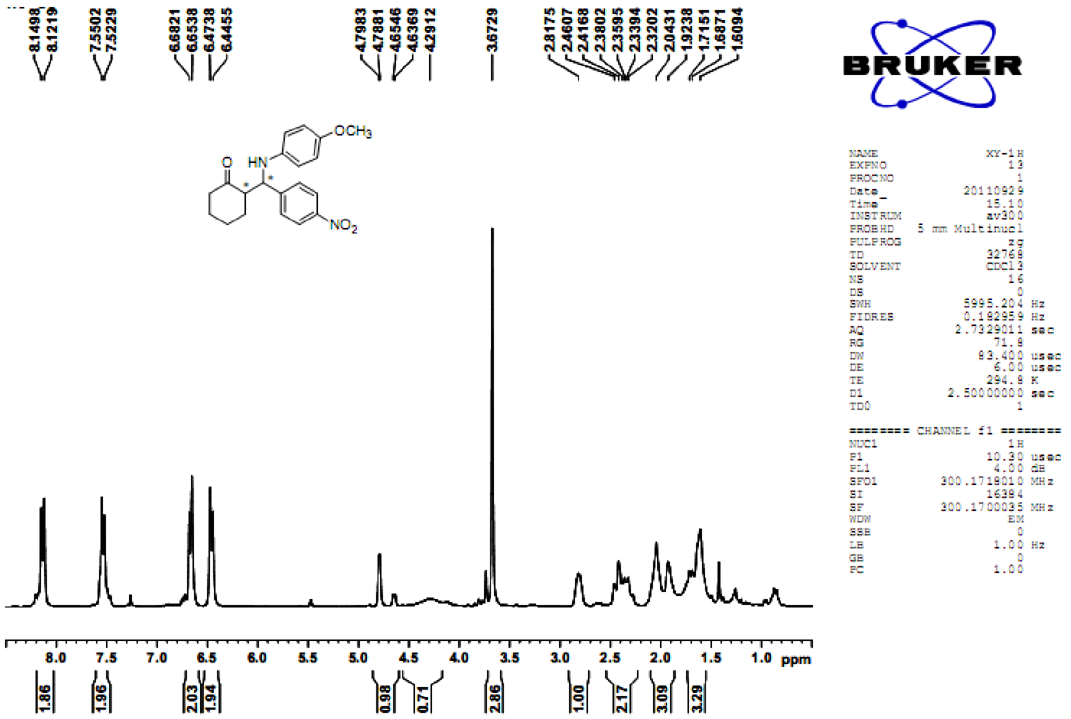

^{13}C NMR Spectrum ($CDCl_3$) of **4n**

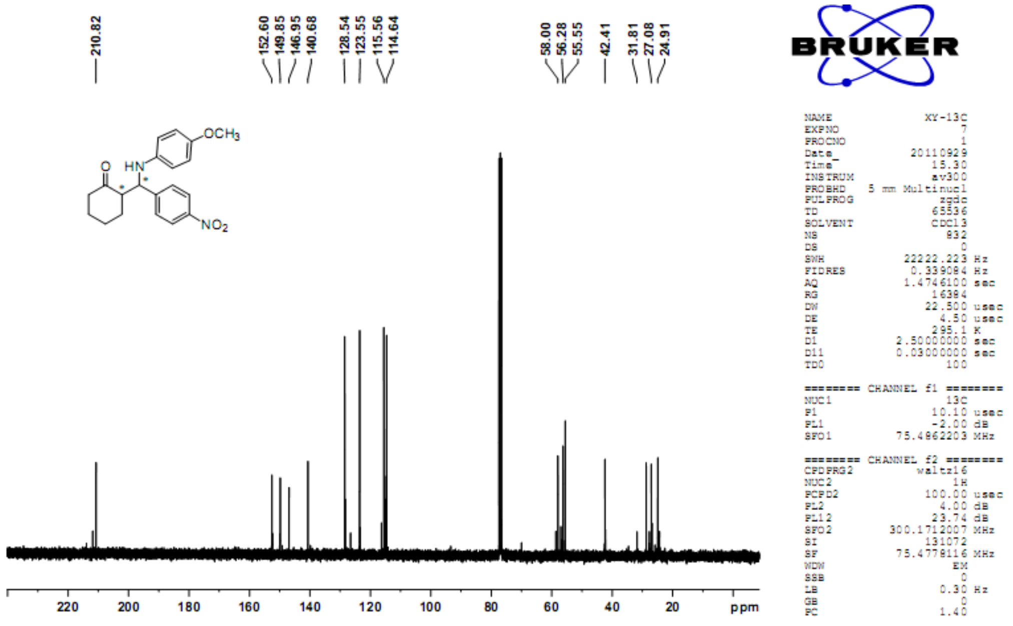

^{1}H NMR Spectrum ($CDCl_3$) of **4o**

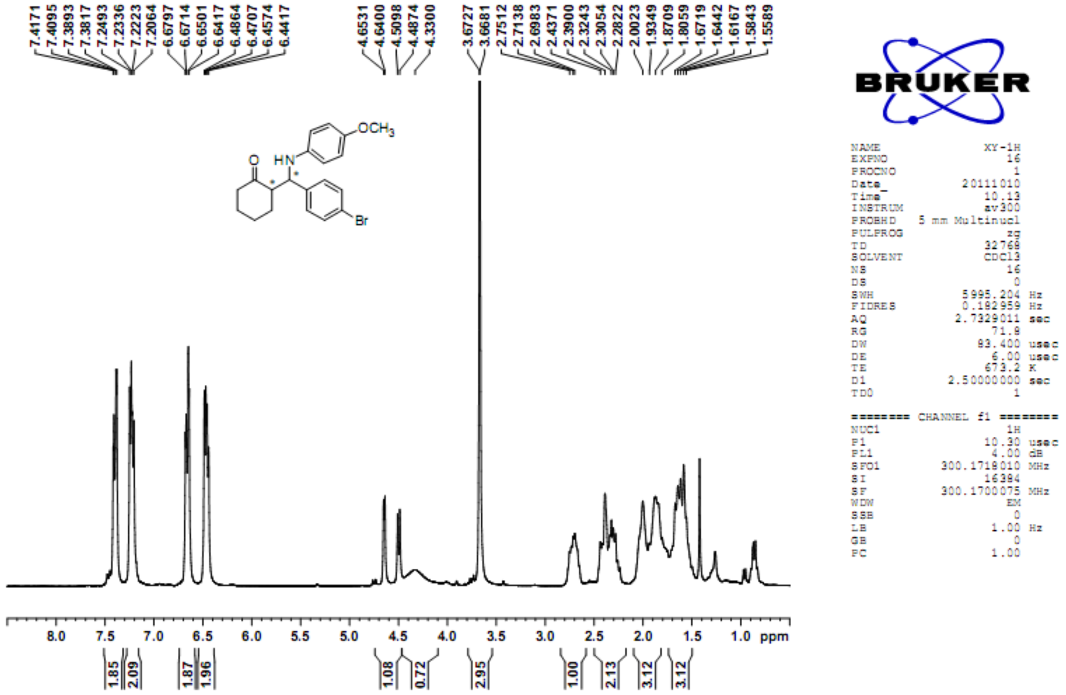

^{13}C NMR Spectrum ($CDCl_3$) of **4o**

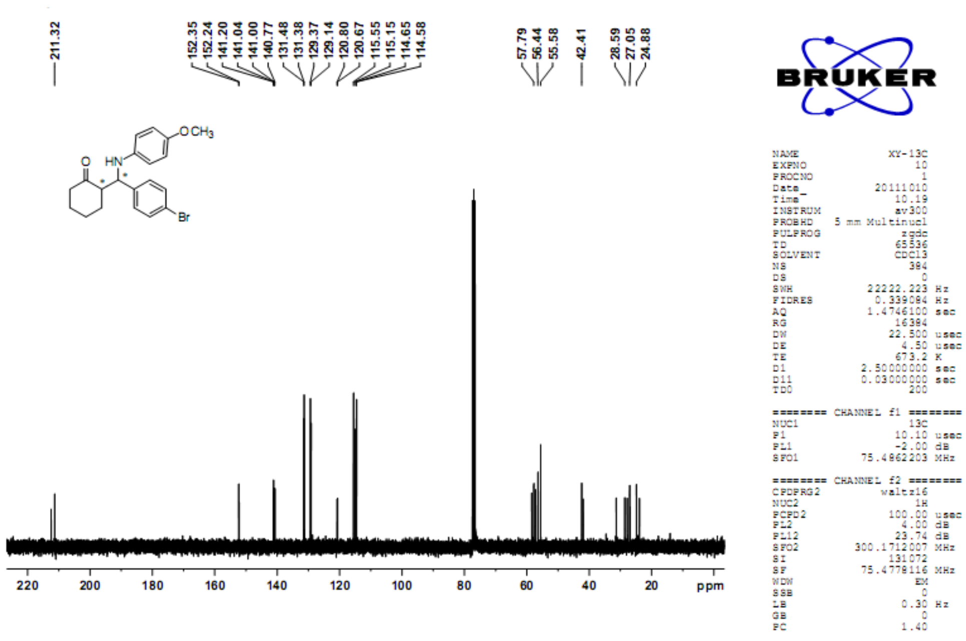

^{1}H NMR Spectrum ($CDCl_3$) of **4p**

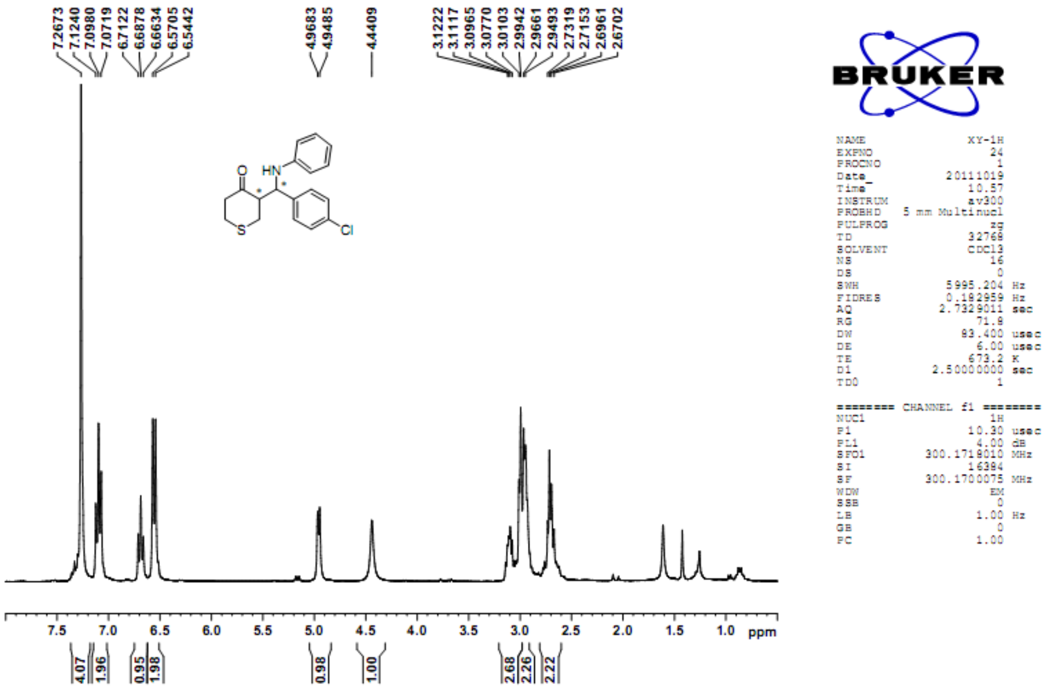

^{13}C NMR Spectrum ($CDCl_3$) of **4p**

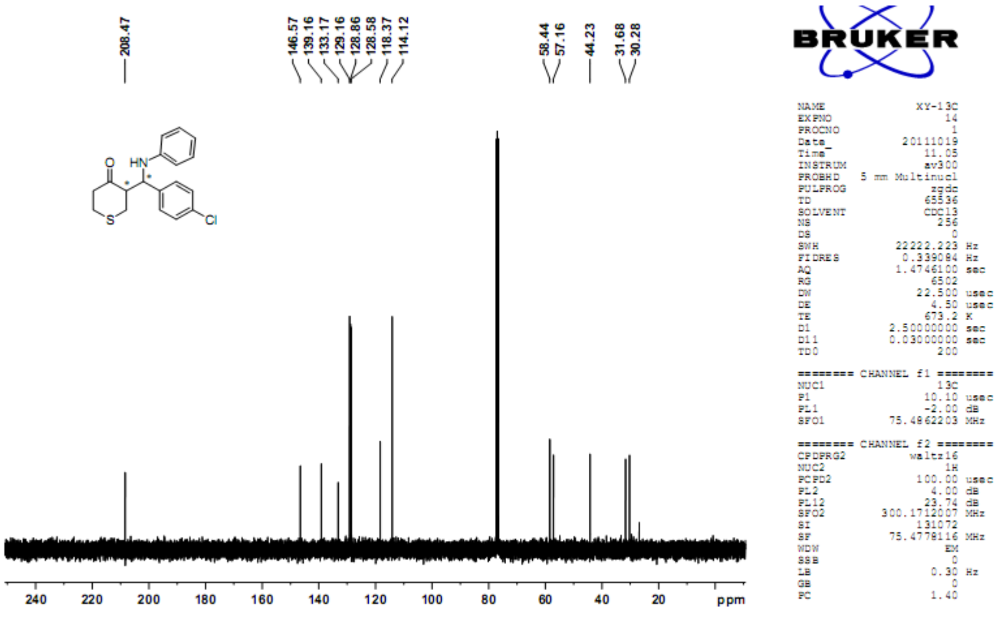

^{1}H NMR Spectrum ($CDCl_3$) of **4q**

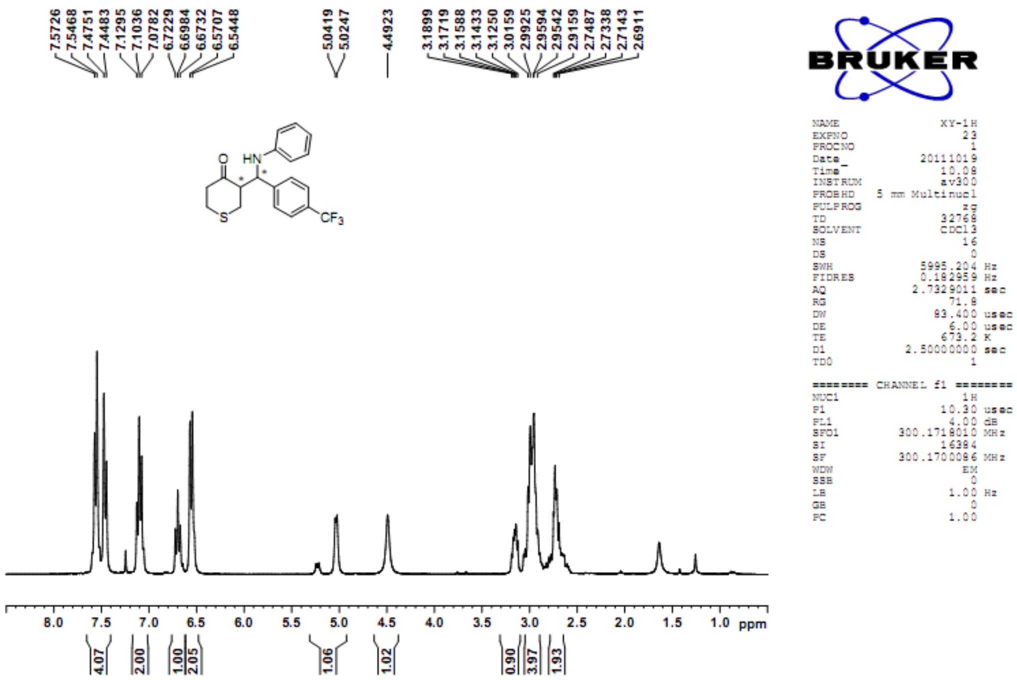

^{13}C NMR Spectrum ($CDCl_3$) of **4q**

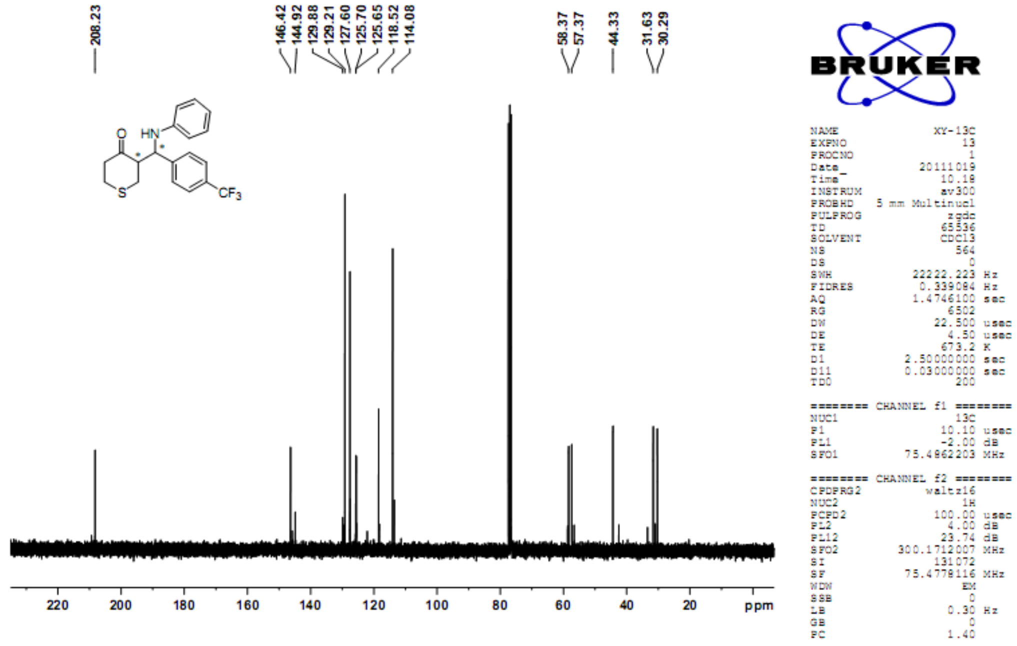

^{1}H NMR Spectrum ($CDCl_3$) of **4r**

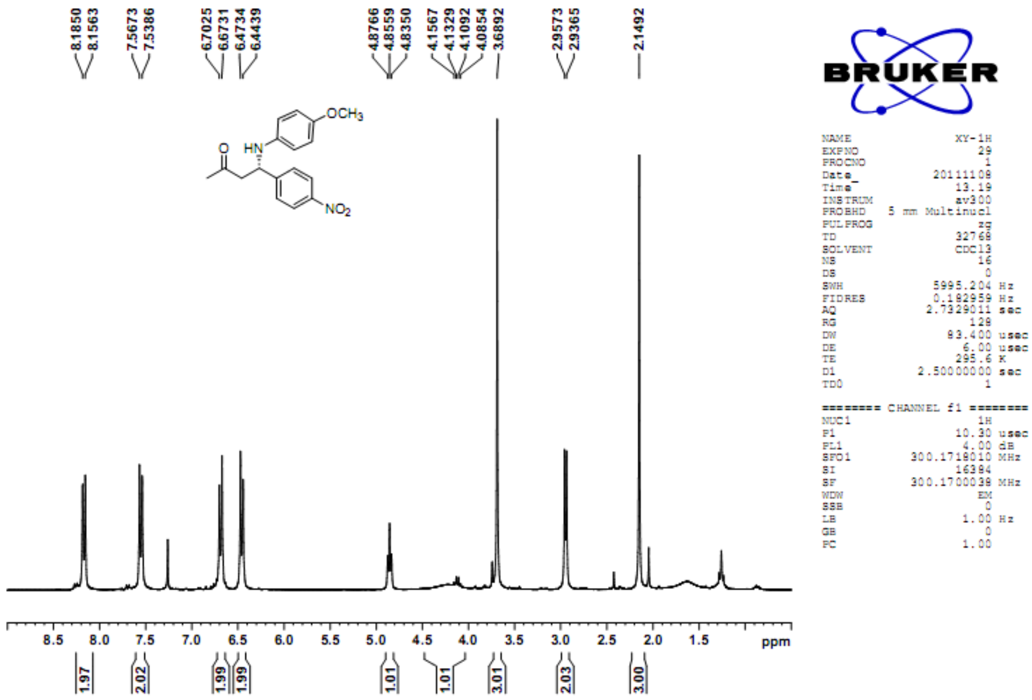

^{13}C NMR Spectrum ($CDCl_3$) of **4r**

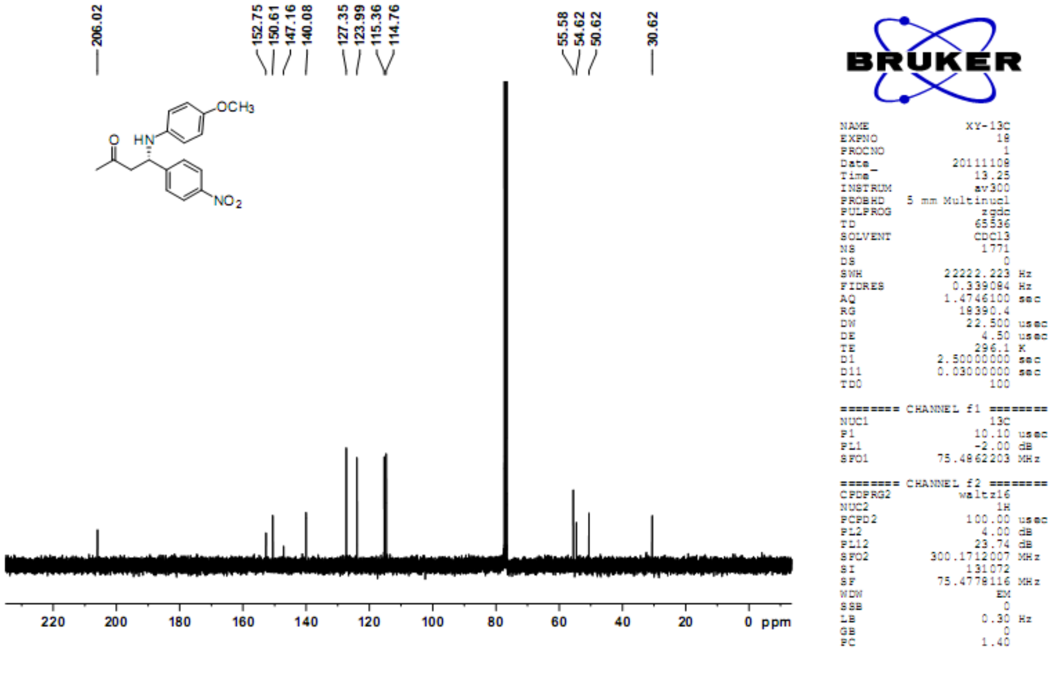

HPLC Spectra of the Mannich Products

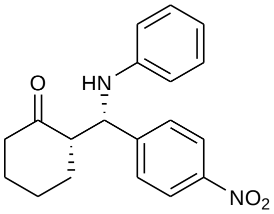

(4a)

4a (Racemic)

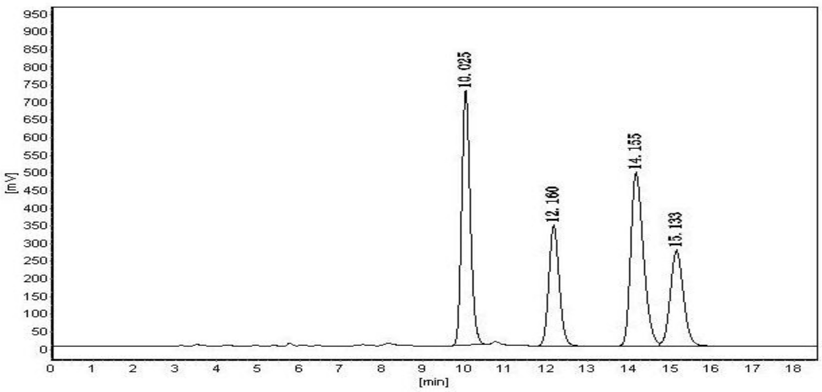

DEFAULT REPORT

Peak #	Time [min]	Height [µv]	Area [µv.s]	Area [%]
1	10.025	718311	10373848	31.66498
2	12.160	339630	5933440	18.11114
3	14.155	489015	10509613	32.07939
4	15.133	268075	5944365	18.14449
Sum		1815031	32761264	100.00000

4a (Chiral)

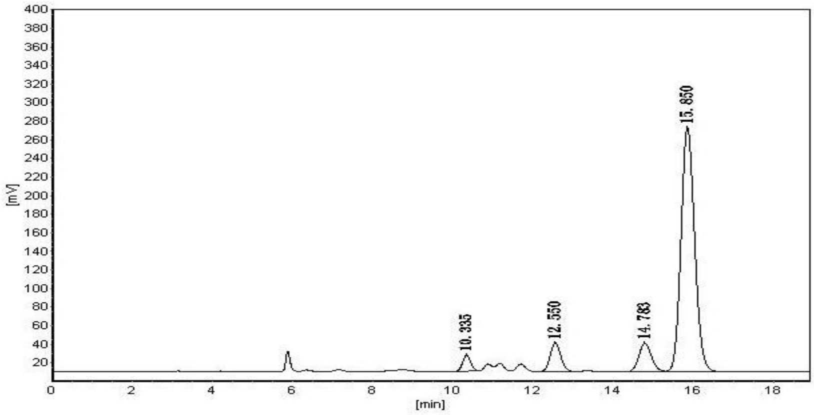

DEFAULT REPORT

Peak #	Time [min]	Height [µv]	Area [µv.s]	Area [%]
1	10.335	16953	240590	3.16769
2	12.550	30720	561597	7.39418
3	14.783	30094	646863	8.51683
4	15.850	262050	6146066	80.92129
Sum		339817	7595116	99.99999

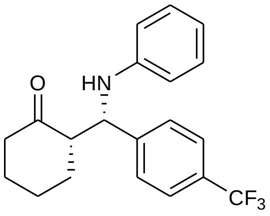

(4b)

4b (Racemic)

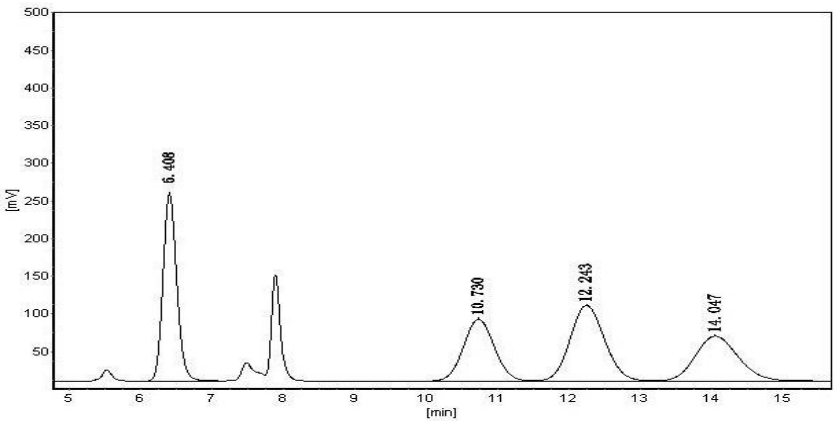

DEFAULT REPORT

Peak #	Time [min]	Height [μv]	Area [μv.s]	Area [%]
1	6.408	247990	3321946	28.43013
2	10.730	81975	2470883	21.14650
3	12.243	100249	3378470	28.91388
4	14.047	59494	2513297	21.50949
Sum		489708	11684596	100.00000

4b (Chiral)

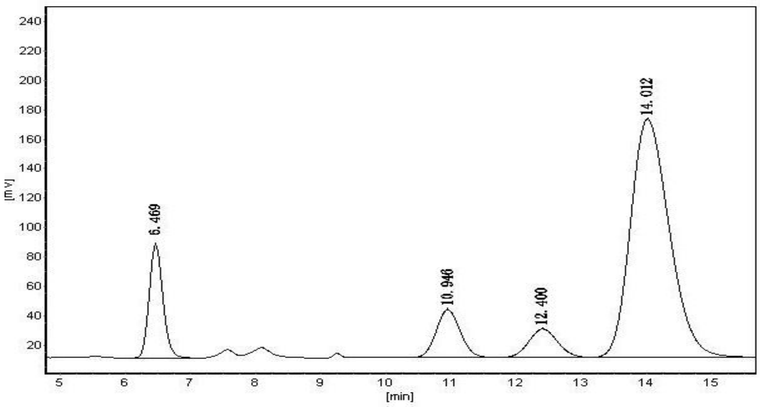

DEFAULT REPORT

Peak #	Time [min]	Height [μv]	Area [μv.s]	Area [%]
1	6.469	76888	1128677	12.17772
2	10.946	32322	826715	8.91975
3	12.400	19272	595229	6.42215
4	14.012	162053	6717752	72.48038
Sum		290535	9268373	100.00000

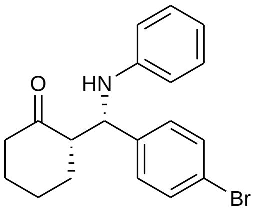

(4c)

4c (Racemic)

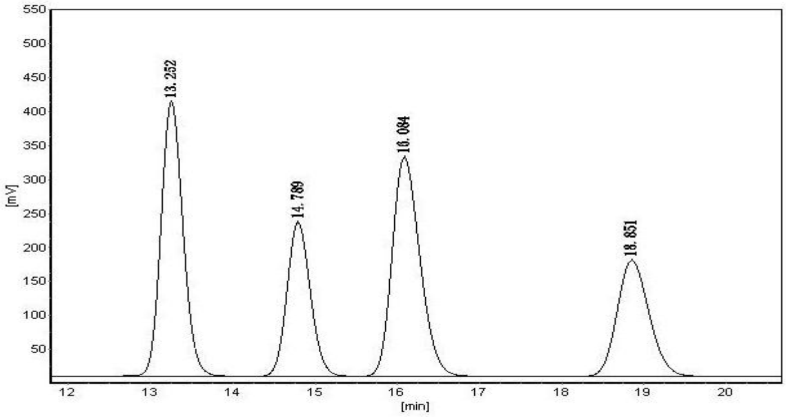

DEFAULT REPORT

Peak #	Time [min]	Height [µv]	Area [µv.s]	Area [%]
1	13.252	404037	7517526	31.05685
2	14.789	226064	4492110	18.55807
3	16.084	321849	7583574	31.32971
4	18.851	170359	4612486	19.05538
Sum		1122309	24205696	100.00002

4c (Chiral)

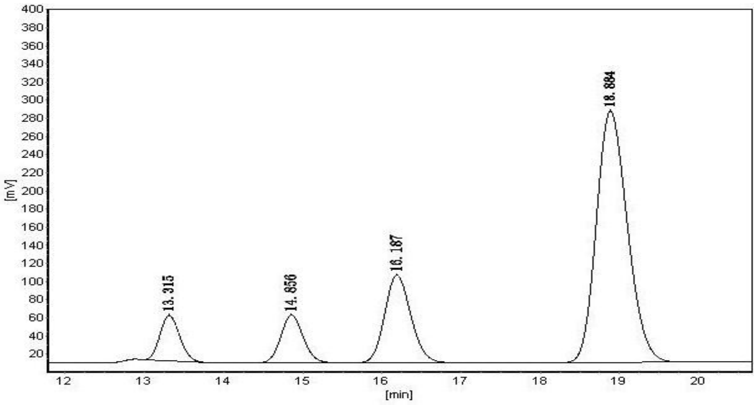

DEFAULT REPORT

Peak #	Time [min]	Height [µv]	Area [µv.s]	Area [%]
1	13.315	49873	865161	7.43178
2	14.856	52685	1032665	8.87065
3	16.187	96487	2213516	19.01422
4	18.884	276630	7530031	64.68336
Sum		475675	11641373	100.00001

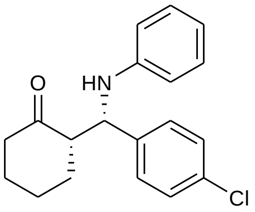

(4d)

4d (Racemic)

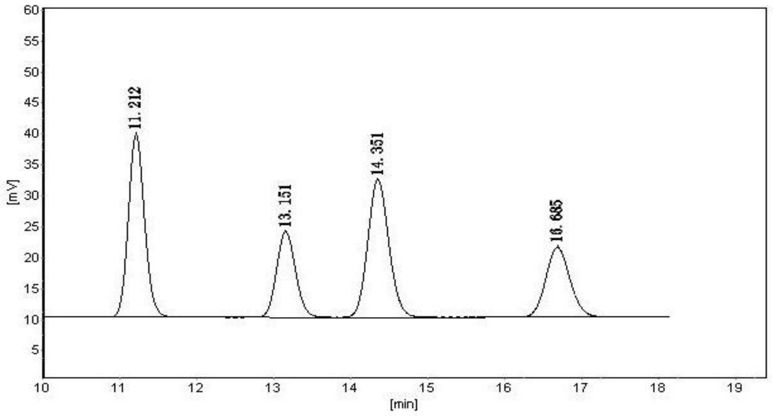

DEFAULT REPORT

Peak #	Time [min]	Height [μv]	Area [μv.s]	Area [%]
1	11.212	29519	431336	32.12468
2	13.151	13883	237820	17.71218
3	14.351	22258	424575	31.62117
4	16.685	11336	248962	18.54197
Sum		76996	1342693	100.00000

4d (Chiral)

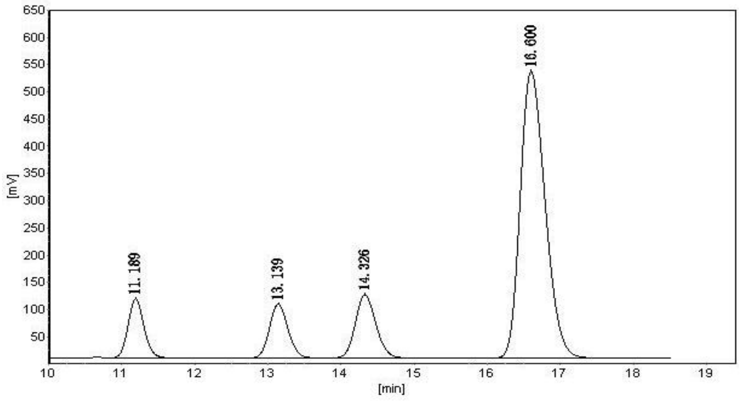

DEFAULT REPORT

Peak #	Time [min]	Height [μv]	Area [μv.s]	Area [%]
1	11.189	108525	1632383	9.01593
2	13.139	99043	1753685	9.68590
3	14.326	116059	2269323	12.53386
4	16.600	527176	12450150	68.76430
Sum		850803	18105540	99.99999

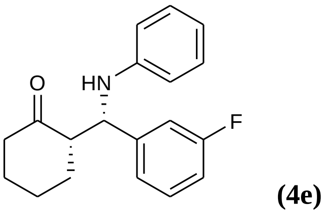

(4e)

4e (Racemic)

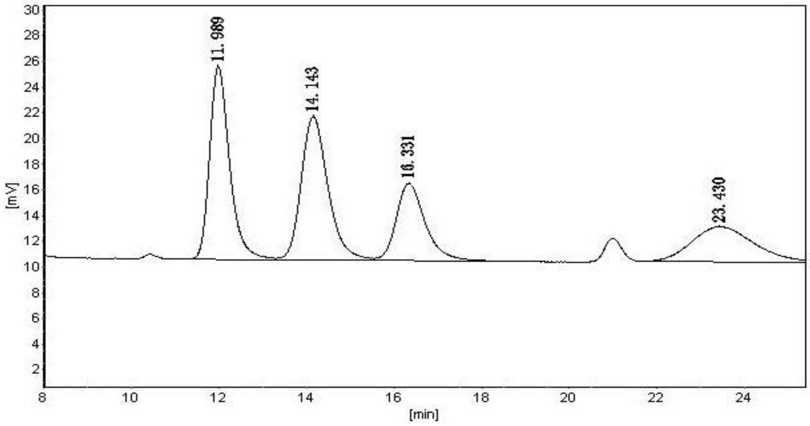

DEFAULT REPORT

Peak #	Time [min]	Height [µv]	Area [µv.s]	Area [%]
1	11.989	15016	476171	31.58665
2	14.143	11170	476151	31.58534
3	16.331	6016	280505	18.60720
4	23.430	2726	274680	18.22082
Sum		34928	1507507	100.00001

4e (Chiral)

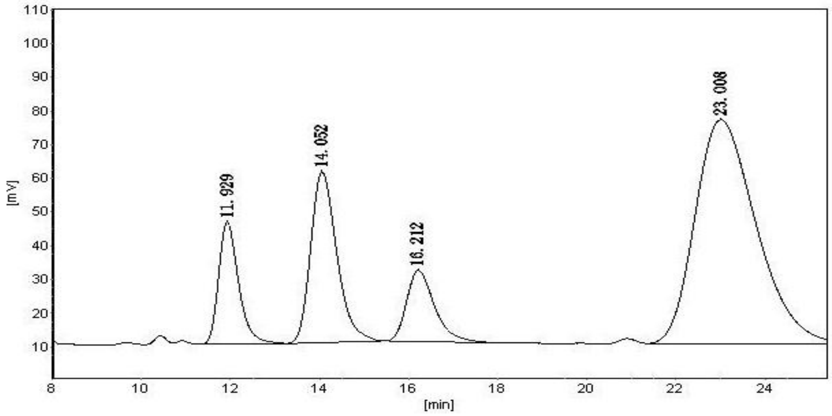

DEFAULT REPORT

Peak #	Time [min]	Height [µv]	Area [µv.s]	Area [%]
1	11.929	36153	1121592	10.72915
2	14.052	50611	2064682	19.75076
3	16.212	21143	930063	8.89699
4	23.008	66471	6337348	60.62310
Sum		174378	10453685	100.00000

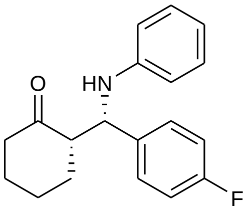

(4f)

4f (Racemic)

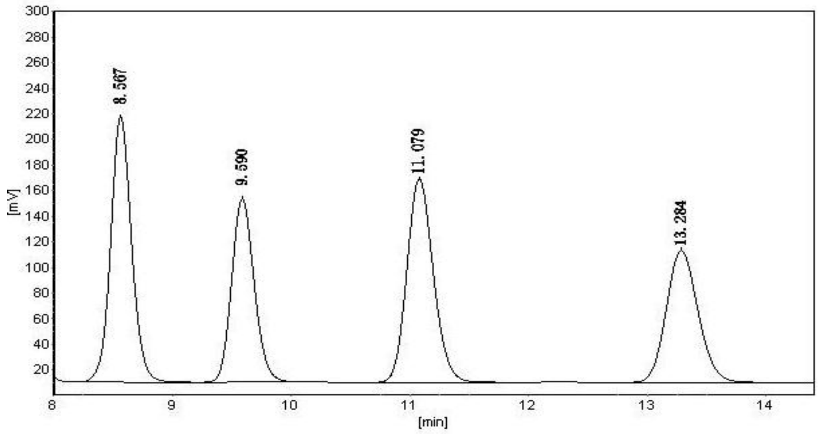

DEFAULT REPORT

Peak #	Time [min]	Height [µv]	Area [µv.s]	Area [%]
1	8.567	207201	2531507	28.78323
2	9.590	143101	1883330	21.41345
3	11.079	159072	2463448	28.00940
4	13.284	103212	1916793	21.79392
Sum		612586	8795078	100.00000

4f (Chiral)

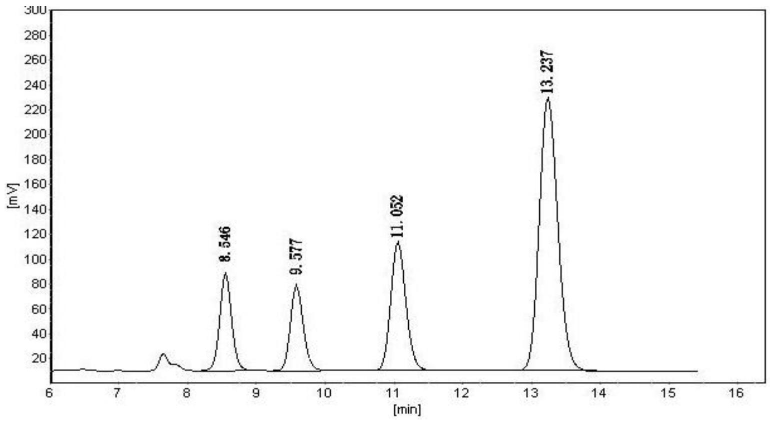

DEFAULT REPORT

Peak #	Time [min]	Height [µv]	Area [µv.s]	Area [%]
1	8.546	77365	949080	12.60276
2	9.577	67685	901717	11.97383
3	11.052	102439	1585643	21.05564
4	13.237	218492	4094290	54.36777
Sum		465981	7530730	100.00000

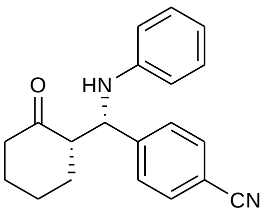

(4g)

4g (Racemic)

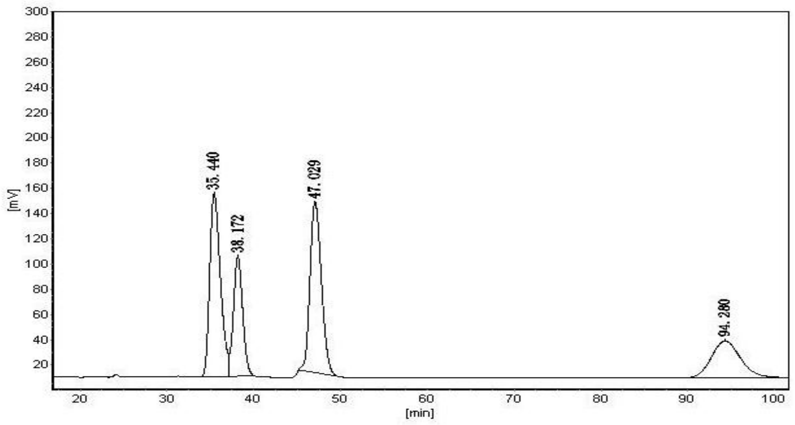

DEFAULT REPORT

Peak #	Time [min]	Height [μv]	Area [μv.s]	Area [%]
1	35.440	145094	12441179	32.25244
2	38.172	94950	7050557	18.27782
3	47.029	135141	12336843	31.98196
4	94.280	29108	6745799	17.48777
Sum		404293	38574380	99.99999

4g (Chiral)

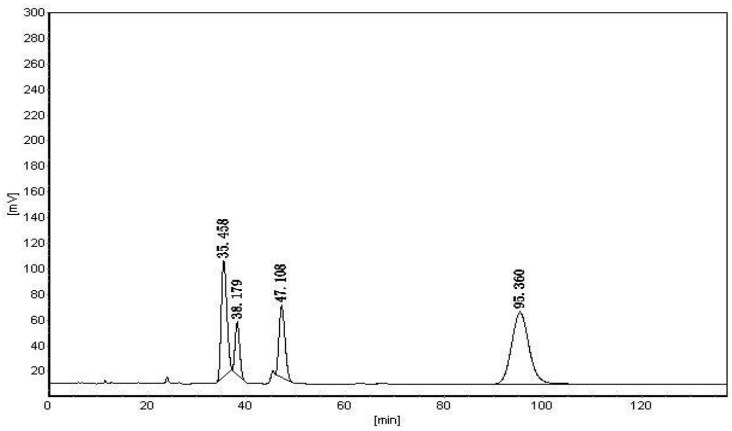

DEFAULT REPORT

Peak #	Time [min]	Height [μv]	Area [μv.s]	Area [%]
1	35.458	89639	7020304	25.18766
2	38.179	40382	2597473	9.31929
3	47.108	55234	4623421	16.58805
4	95.360	55649	13630799	48.90499
Sum		240904	27871996	99.99999

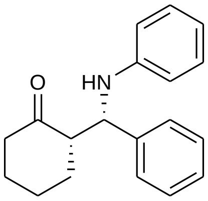

(4h)

4h (Racemic)

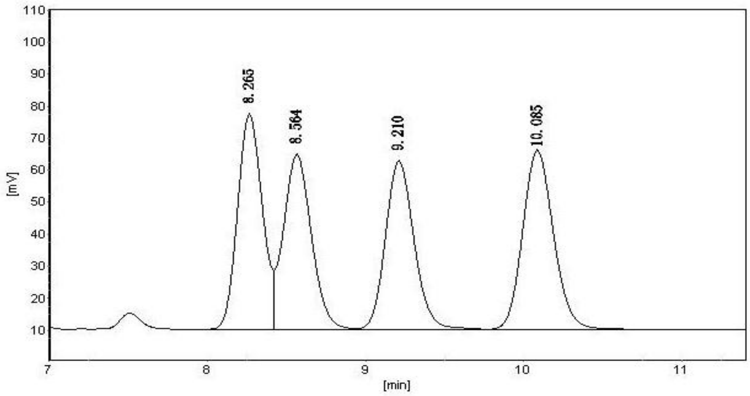

DEFAULT REPORT

Peak #	Time [min]	Height [µv]	Area [µv.s]	Area [%]
1	8.265	67119	730458	25.87990
2	8.564	54511	670196	23.74481
3	9.210	52577	661452	23.43504
4	10.085	55922	760387	26.94025
Sum		230129	2822493	100.00000

4h (Chiral)

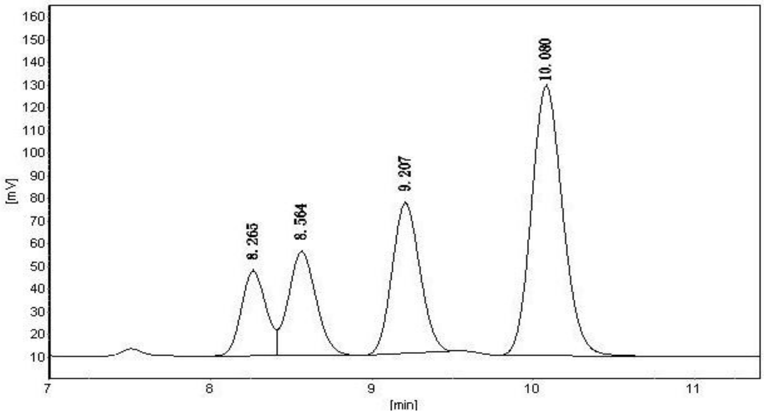

DEFAULT REPORT

Peak #	Time [min]	Height [µv]	Area [µv.s]	Area [%]
1	8.265	37223	396154	11.87336
2	8.564	45889	550970	16.51342
3	9.207	66163	792399	23.74943
4	10.080	118891	1596973	47.86378
Sum		268166	3336496	99.99999

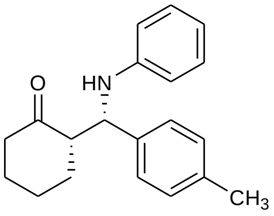

(4i)

4i (Racemic)

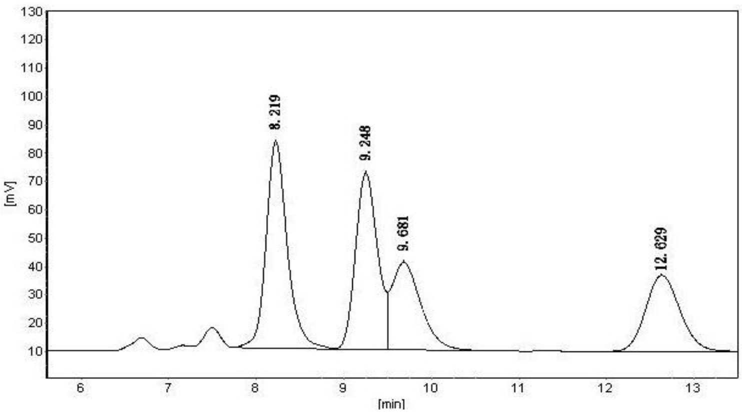

DEFAULT REPORT

Peak #	Time [min]	Height [µv]	Area [µv.s]	Area [%]
1	8.219	72923	1226562	32.54041
2	9.248	62190	1091929	28.96865
3	9.681	30868	715369	18.97860
4	12.629	26898	735488	19.51234
Sum		192879	3769348	100.00000

4i (Chiral)

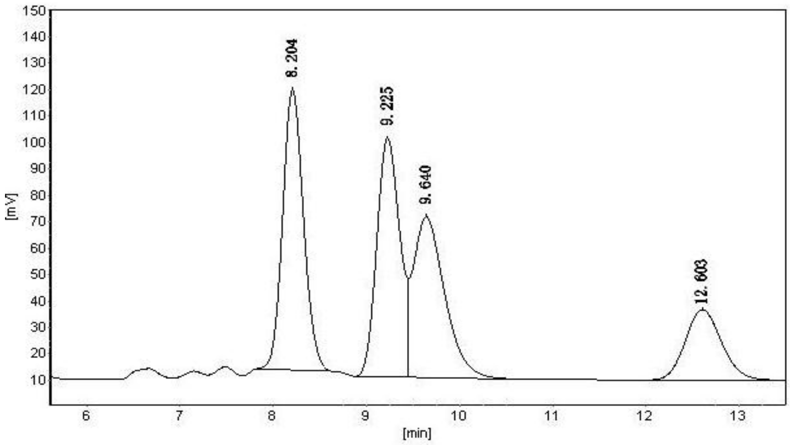

DEFAULT REPORT

Peak #	Time [min]	Height [µv]	Area [µv.s]	Area [%]
1	8.204	106127	1650906	30.76473
2	9.225	90472	1564399	29.15268
3	9.640	60940	1424586	26.54726
4	12.603	26721	726337	13.53533
Sum		284260	5366228	100.00000

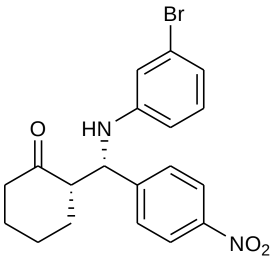

(4j)

4j (Racemic)

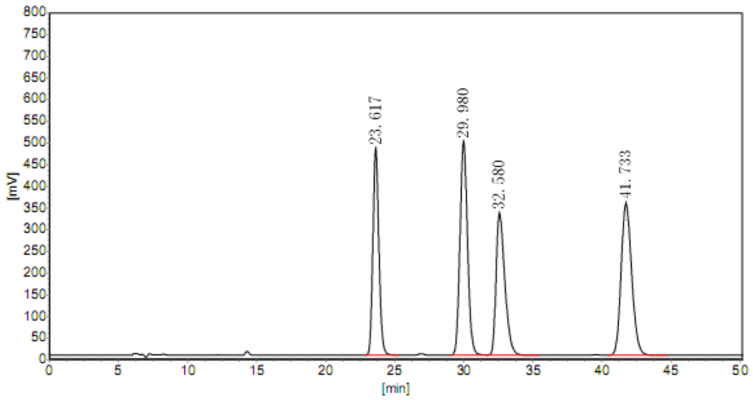

DEFAULT REPORT

Peak #	Time [min]	Height [µv]	Area [µv.s]	Area [%]
1	23.617	476322	14358770	21.71917
2	29.980	491558	18633639	28.18536
3	32.580	326236	14465694	21.88090
4	41.733	349303	18652944	28.21457
Sum		1643419	66111048	100.00000

4j (Chiral)

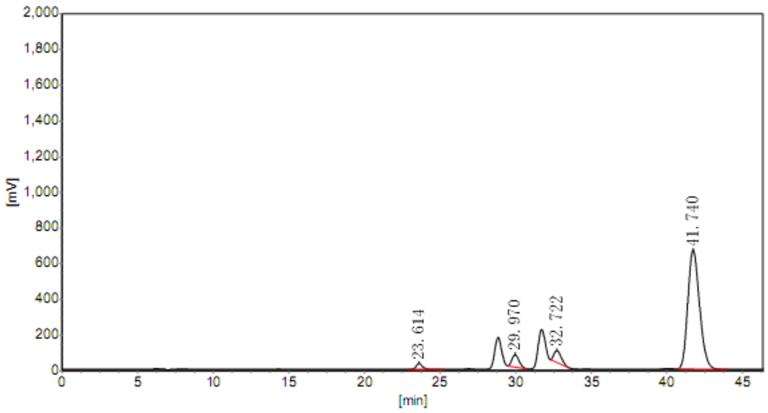

DEFAULT REPORT

Peak #	Time [min]	Height [µv]	Area [µv.s]	Area [%]
1	23.614	31466	948609	2.29959
2	29.970	68096	2244195	5.44030
3	32.722	65708	2247903	5.44929
4	41.740	663880	35810609	86.81083
Sum		829150	41251316	100.00001

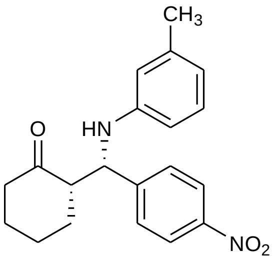

(4k)

4k (Racemic)

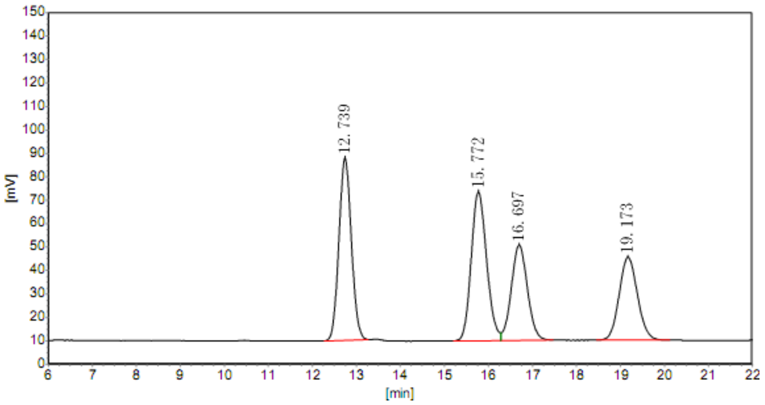

DEFAULT REPORT

Peak #	Time [min]	Height [μv]	Area [μv.s]	Area [%]
1	12.739	77647	1528983	29.77000
2	15.772	63366	1546436	30.10982
3	16.697	40504	1032859	20.11024
4	19.173	35393	1027707	20.00993
Sum		216910	5135985	99.99999

4k (Chiral)

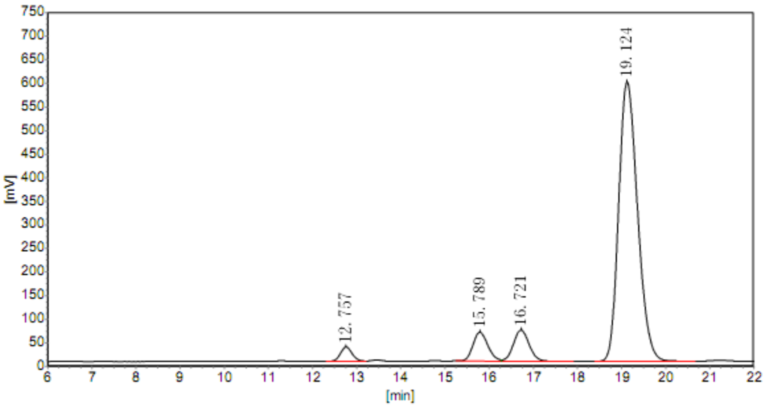

DEFAULT REPORT

Peak #	Time [min]	Height [μv]	Area [μv.s]	Area [%]
1	12.757	30075	553403	2.60886
2	15.789	61106	1438632	6.78203
3	16.721	66194	1632674	7.69679
4	19.124	592465	17587685	82.91231
Sum		749840	21212394	99.99999

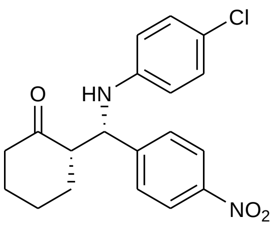

(4l)

4l (Racemic)

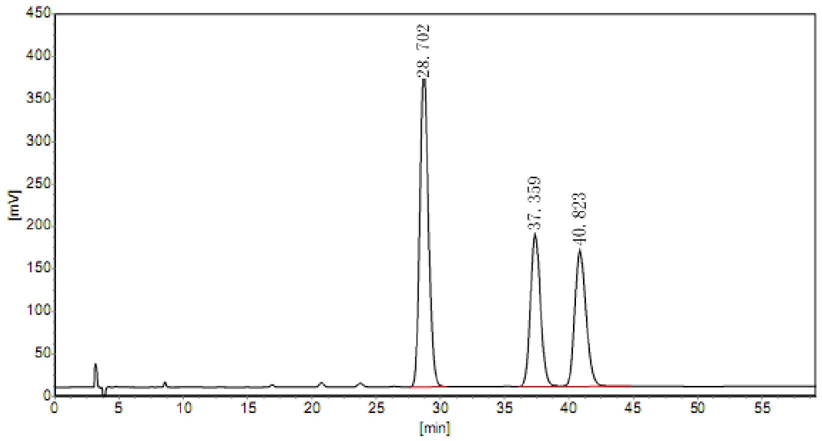

DEFAULT REPORT

Peak #	Time [min]	Height [µv]	Area [µv.s]	Area [%]
1	28.702	369712	17262435	46.90993
2	37.359	177577	9723809	26.42403
3	40.823	159189	9812865	26.66604
Sum		706478	36799108	100.00000

4l (Chiral)

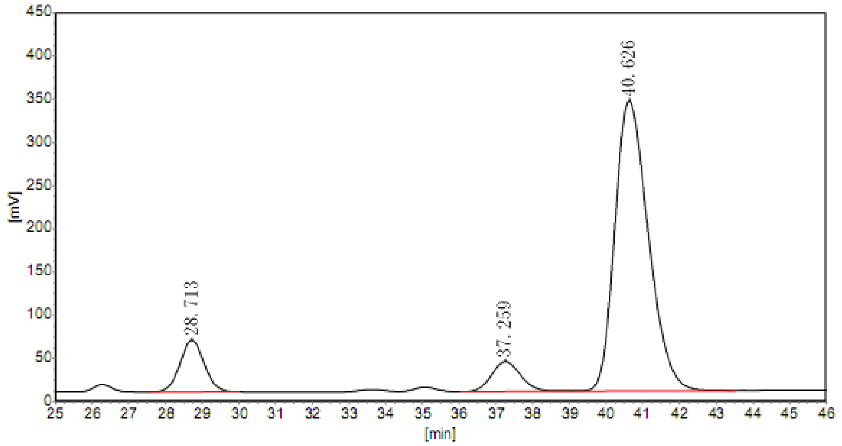

DEFAULT REPORT

Peak #	Time [min]	Height [µv]	Area [µv.s]	Area [%]
1	28.713	59725	2754377	10.59484
2	37.259	34597	1921857	7.39251
3	40.626	335718	21321103	82.01264
Sum		430040	25997336	99.99999

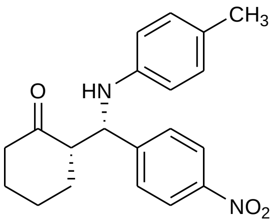

(4m)

4m (Racemic)

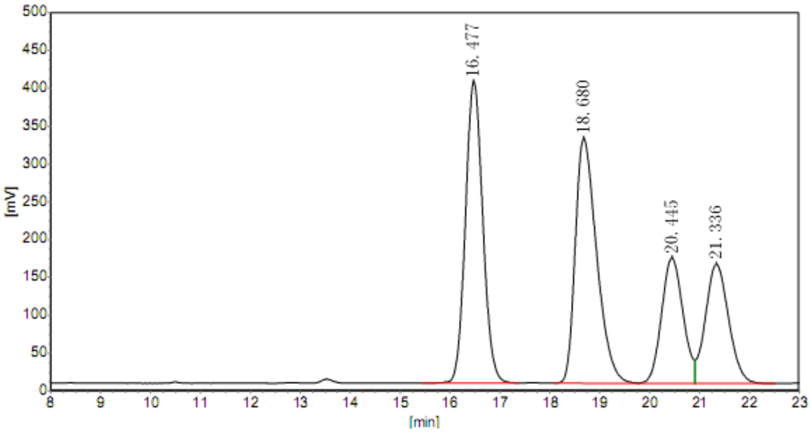

DEFAULT REPORT

Peak #	Time [min]	Height [μv]	Area [μv.s]	Area [%]
1	16.477	398435	9637577	32.81233
2	18.680	323420	9652661	32.86369
3	20.445	165958	4973071	16.93144
4	21.336	157828	5108505	17.39254
Sum		1045641	29371812	100.00000

4m (Chiral)

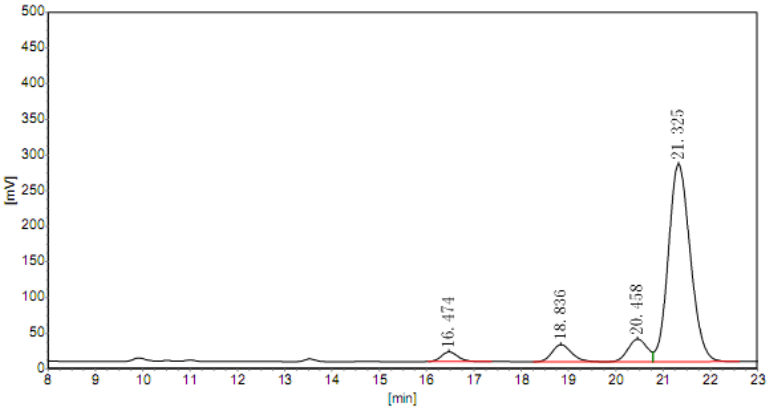

DEFAULT REPORT

Peak #	Time [min]	Height [μv]	Area [μv.s]	Area [%]
1	16.474	13609	335718	3.08543
2	18.836	24167	701653	6.44856
3	20.458	31782	896874	8.24275
4	21.325	276933	8946520	82.22326
Sum		346491	10880765	100.00000

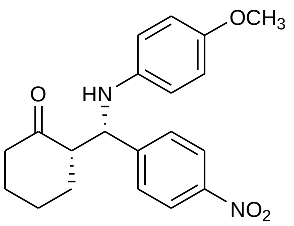

(4n)

4n (Racemic)

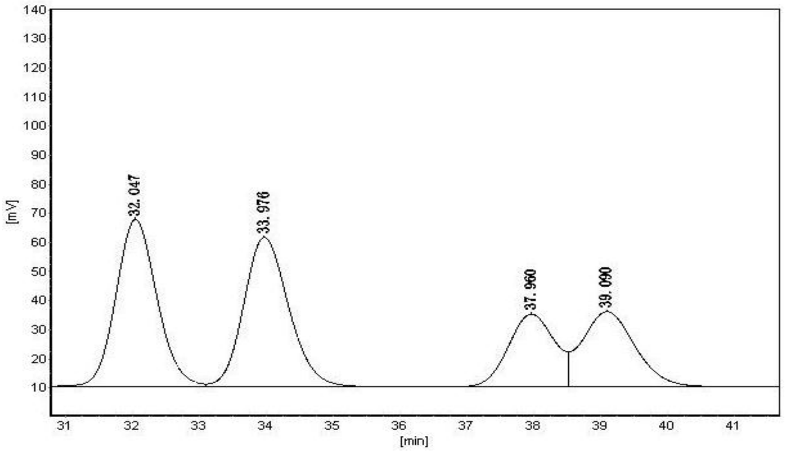

DEFAULT REPORT

Peak #	Time [min]	Height [µv]	Area [µv.s]	Area [%]
1	32.047	57340	2433995	32.66836
2	33.976	51266	2377281	31.90716
3	37.960	24935	1201438	16.12535
4	39.090	25803	1437905	19.29913
Sum		159344	7450619	100.00001

4n (Chiral)

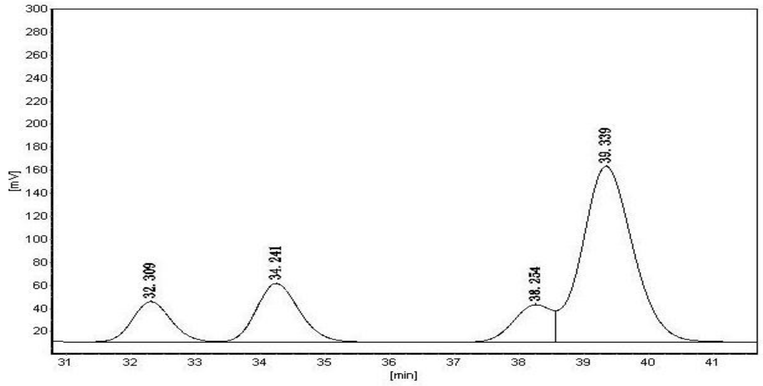

DEFAULT REPORT

Peak #	Time [min]	Height [µv]	Area [µv.s]	Area [%]
1	32.309	34778	1445660	10.71373
2	34.241	50791	2329160	17.26132
3	38.254	32340	1317134	9.76123
4	39.339	152809	8401567	62.26371
Sum		270718	13493521	99.99998

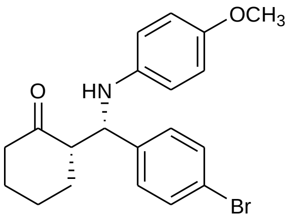

(4o)

4o (Racemic)

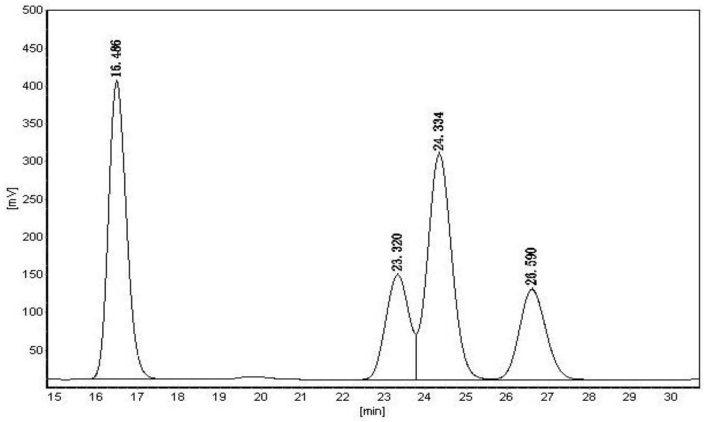

DEFAULT REPORT

Peak #	Time [min]	Height [µv]	Area [µv.s]	Area [%]
1	16.486	393573	11895880	33.96534
2	23.320	138485	5465496	15.60519
3	24.334	298552	12283393	35.07177
4	26.590	119401	5378820	15.35771
Sum		950011	35023588	100.00002

4o (Chiral)

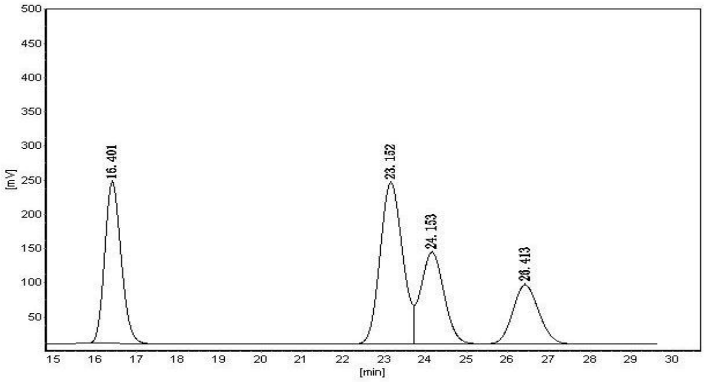

DEFAULT REPORT

Peak #	Time [min]	Height [µv]	Area [µv.s]	Area [%]
1	16.401	235487	6504277	26.45661
2	23.152	235789	8969277	36.48317
3	24.153	134013	5291639	21.52412
4	26.413	86488	3819506	15.53611
Sum		691777	24584698	100.00001

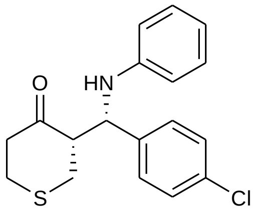

(4p)

4p (Racemic)

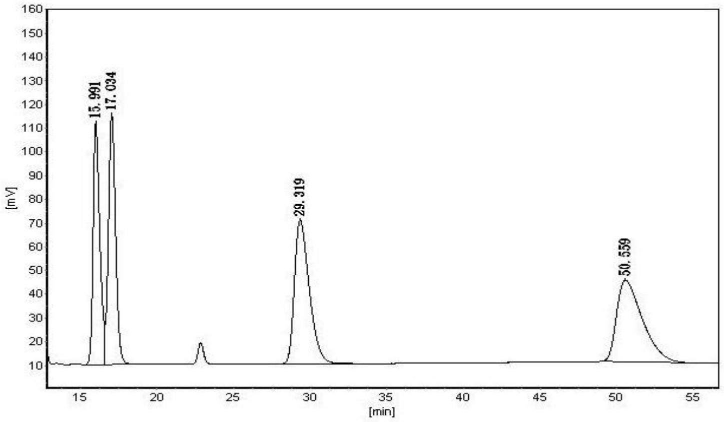

DEFAULT REPORT

Peak #	Time [min]	Height [µv]	Area [µv.s]	Area [%]
1	15.991	101894	3093355	21.10805
2	17.034	105593	3429231	23.39996
3	29.319	60565	4110844	28.05107
4	50.559	34367	4021427	27.44092
Sum		302419	14654857	100.00000

4p (Chiral)

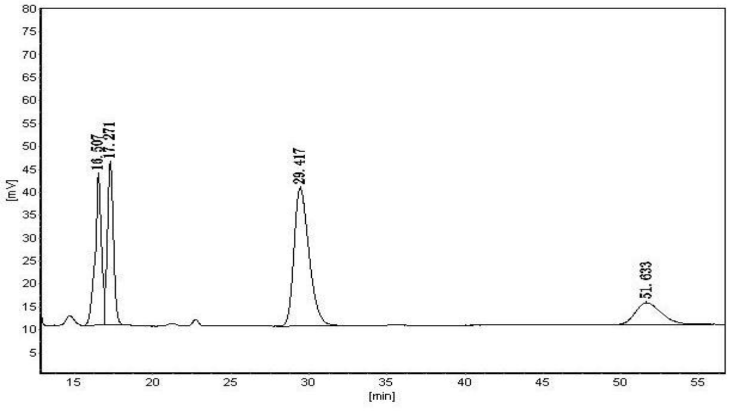

DEFAULT REPORT

Peak #	Time [min]	Height [µv]	Area [µv.s]	Area [%]
1	16.507	32864	934355	20.95176
2	17.271	35439	951016	21.32536
3	29.417	30072	2015397	45.19281
4	51.633	4802	558785	12.53008
Sum		103177	4459553	100.00001

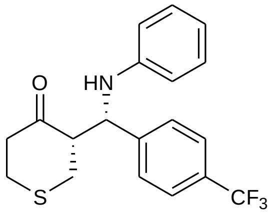

(4q)

4q (Racemic)

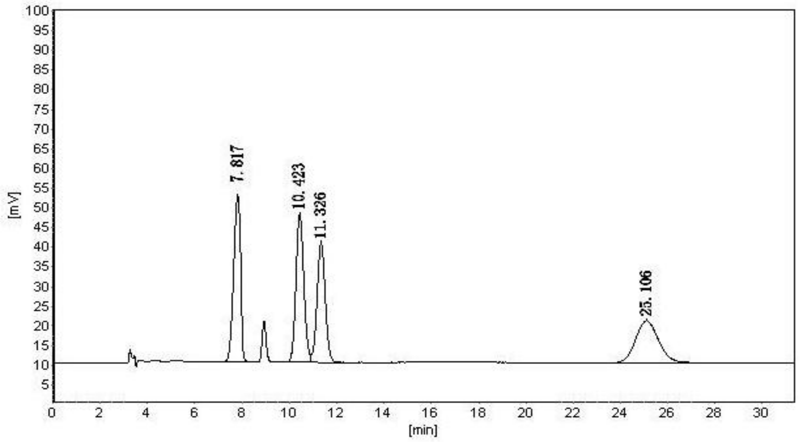

DEFAULT REPORT

Peak #	Time [min]	Height [µv]	Area [µv.s]	Area [%]
1	7.817	42289	871588	26.96408
2	10.423	37703	876616	27.11962
3	11.326	30244	730502	22.59933
4	25.106	10707	753699	23.31697
Sum		120943	3232405	100.00000

4q (Chiral)

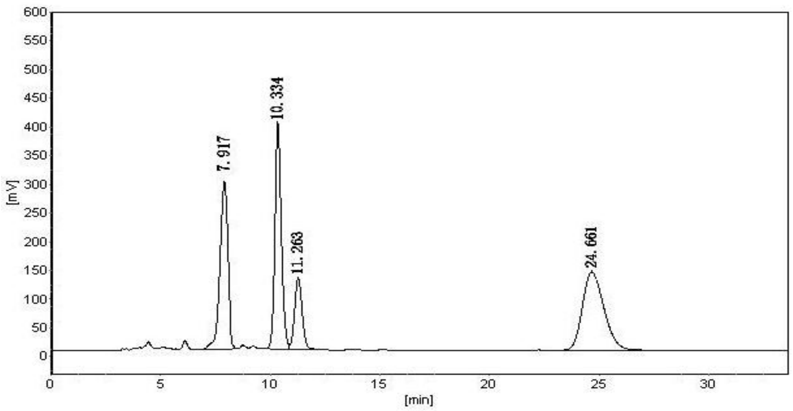

DEFAULT REPORT

Peak #	Time [min]	Height [µv]	Area [µv.s]	Area [%]
1	7.917	289899	7469593	26.94582
2	10.334	392337	7997094	28.84872
3	11.263	123489	2997134	10.81186
4	24.661	136038	9256969	33.39360
Sum		941763	27720788	100.00000

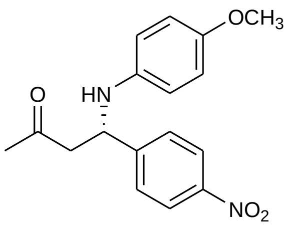

(4r)

4r (Racemic)

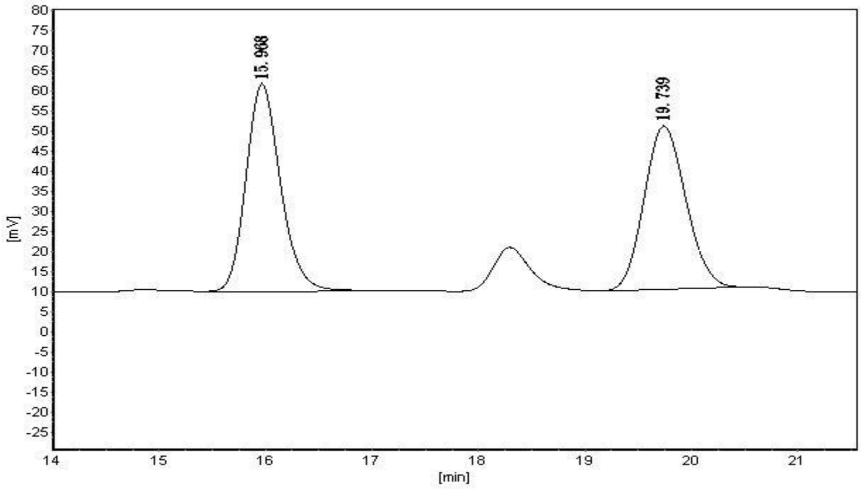

DEFAULT REPORT

Peak #	Time [min]	Height [µv]	Area [µv.s]	Area [%]
1	15.968	51552	1165051	51.29399
2	19.739	40610	1106269	48.70601
Sum		92162	2271320	100.00000

4r (Chiral)

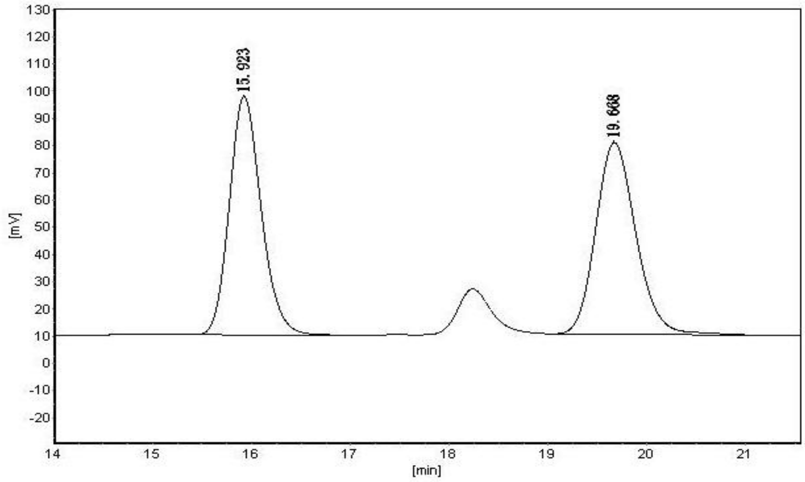

DEFAULT REPORT

Peak #	Time [min]	Height [µv]	Area [µv.s]	Area [%]
1	15.923	87670	1962848	49.59703
2	19.668	70503	1994744	50.40297
Sum		158173	3957592	100.00000

5. HPLC spectra of reaction profiles of the SGP-catalysed the Mannich reaction and the blank reaction.

5.1 HPLC Spectra of Table S1

Entry 3

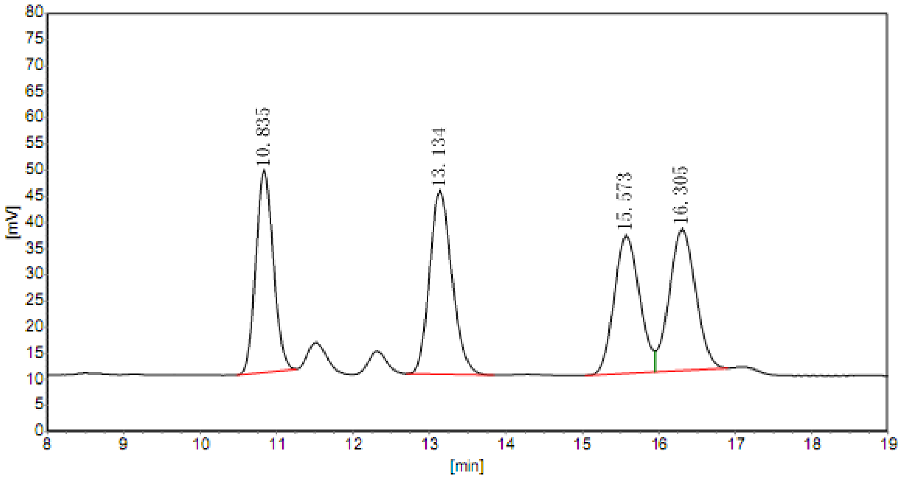

DEFAULT REPORT

Peak #	Time [min]	Height [µv]	Area [µv.s]	Area [%]
1	10.835	38301	614472	23.73462
2	13.134	34832	714991	27.61728
3	15.573	26207	608182	23.49167
4	16.305	26875	651281	25.15643
Sum		126215	2588926	100.00000

Entry 4

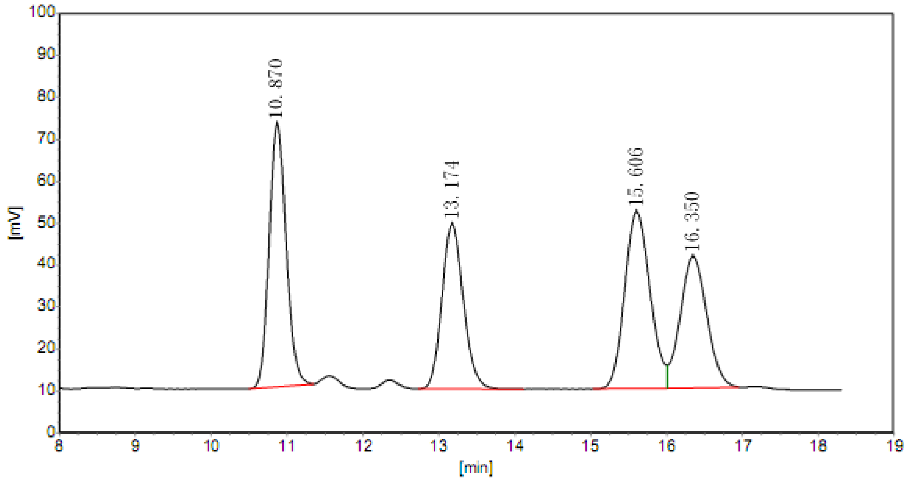

DEFAULT REPORT

Peak #	Time [min]	Height [µv]	Area [µv.s]	Area [%]
1	10.870	62598	975750	27.96939
2	13.174	39194	773259	22.16509

3	15.606	42013	978450	28.04678
4	16.350	31192	761176	21.81874
Sum		174997	3488635	100.00000

Entry 5

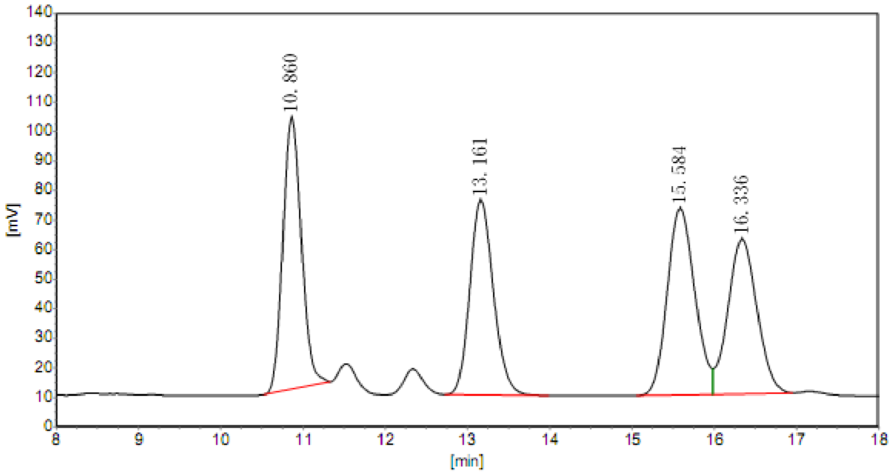

DEFAULT REPORT

Peak #	Time [min]	Height [µv]	Area [µv.s]	Area [%]
1	10.860	91877	1438408	26.08317
2	13.161	65630	1318268	23.90462
3	15.584	62879	1471347	26.68047
4	16.336	52215	1286675	23.33174
Sum		271601	5514698	100.00000

Entry 6

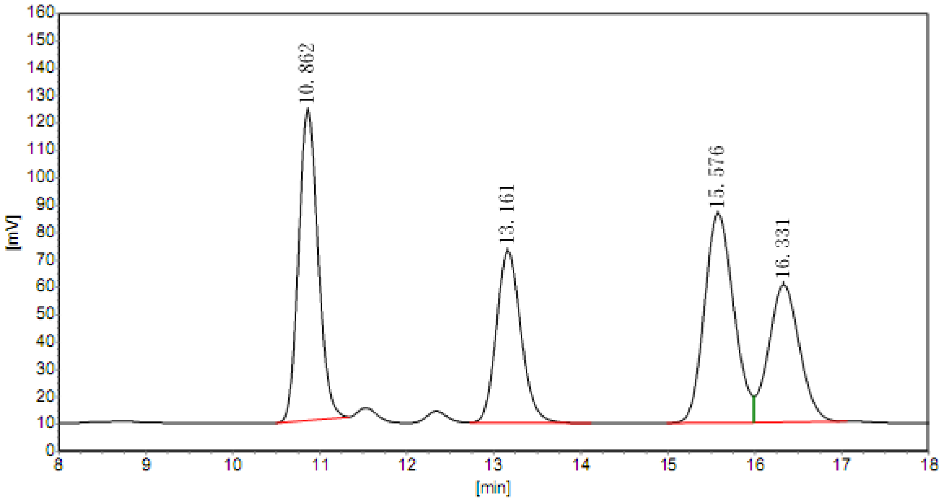

DEFAULT REPORT

Peak #	Time [min]	Height [µv]	Area [µv.s]	Area [%]
1	10.862	113279	1769837	29.31051
2	13.161	62805	1240257	20.54007

3	15.576	76337	1790696	29.65596
4	16.331	50195	1237443	20.49346
Sum		302616	6038233	100.00000

Entry 7

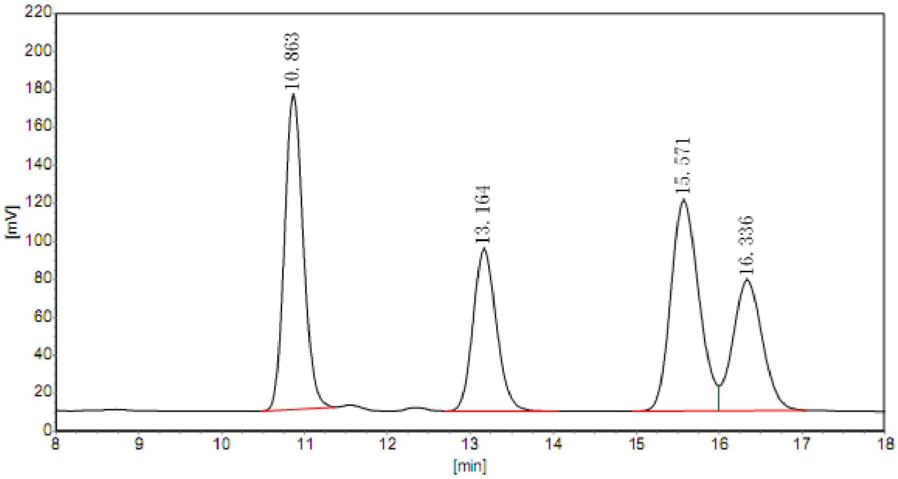

DEFAULT REPORT

Peak #	Time [min]	Height [µv]	Area [µv.s]	Area [%]
1	10.863	164853	2601618	30.33360
2	13.164	84980	1673750	19.51511
3	15.571	110591	2606794	30.39395
4	16.336	68431	1694525	19.75734
Sum		428855	8576687	100.00000

Entry 8

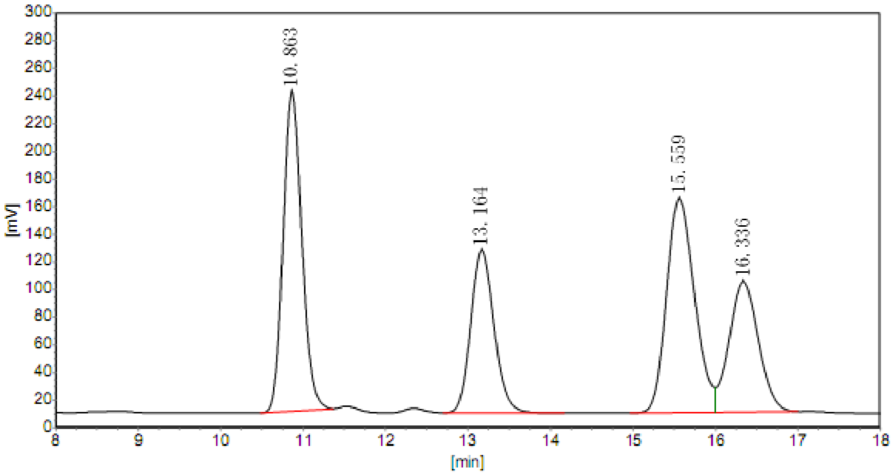

DEFAULT REPORT

Peak #	Time [min]	Height [µv]	Area [µv.s]	Area [%]
1	10.863	231179	3642592	30.47445
2	13.164	117576	2323283	19.43691

3	15.559	154578	3651321	30.54747
4	16.336	94335	2335744	19.54117
Sum		597668	11952940	99.99999

Entry 9

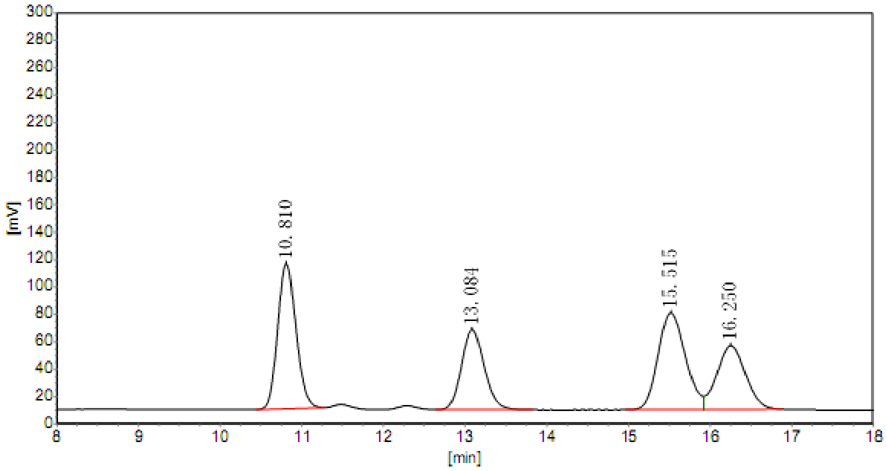

DEFAULT REPORT

Peak #	Time [min]	Height [µv]	Area [µv.s]	Area [%]
1	10.810	106006	1655690	29.51238
2	13.084	58464	1142249	20.36039
3	15.515	70859	1661479	29.61557
4	16.250	46843	1150736	20.51167
Sum		282172	5610154	100.00002

5.2 HPLC Spectra of Table S2

Entry 1

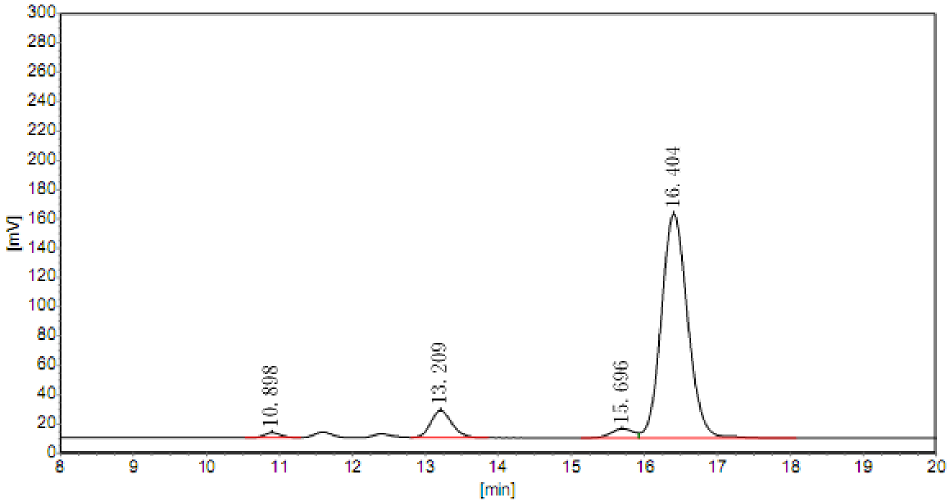

DEFAULT REPORT

Peak #	Time [min]	Height [µv]	Area [µv.s]	Area [%]
1	10.898	3390	52435	1.19344
2	13.209	18358	371030	8.44474

3	15.696	6289	135794	3.09071
4	16.404	152843	3834357	87.27110
Sum		180880	4393616	99.99999

Entry 2

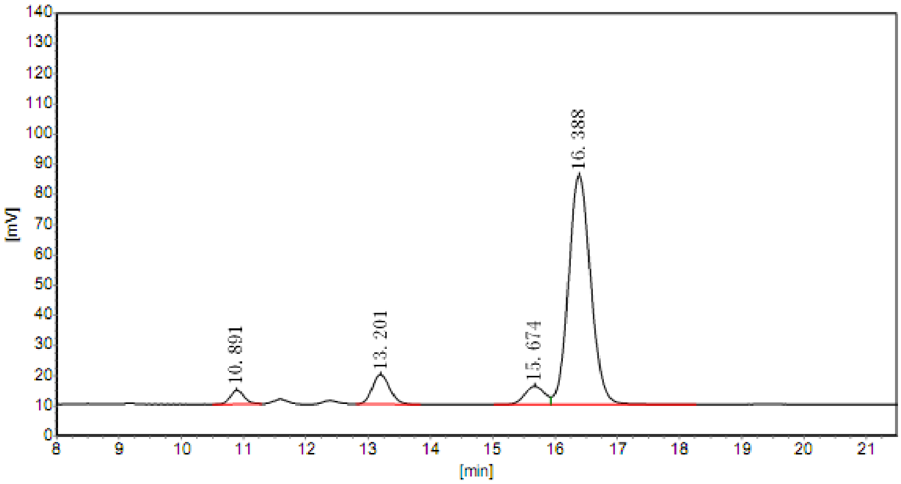

DEFAULT REPORT

Peak #	Time [min]	Height [μv]	Area [μv.s]	Area [%]
1	10.891	4473	69234	3.04916
2	13.201	9781	190431	8.38679
3	15.674	6056	134665	5.93080
4	16.388	75837	1876274	82.63325
Sum		96147	2270604	100.00000

Entry 3

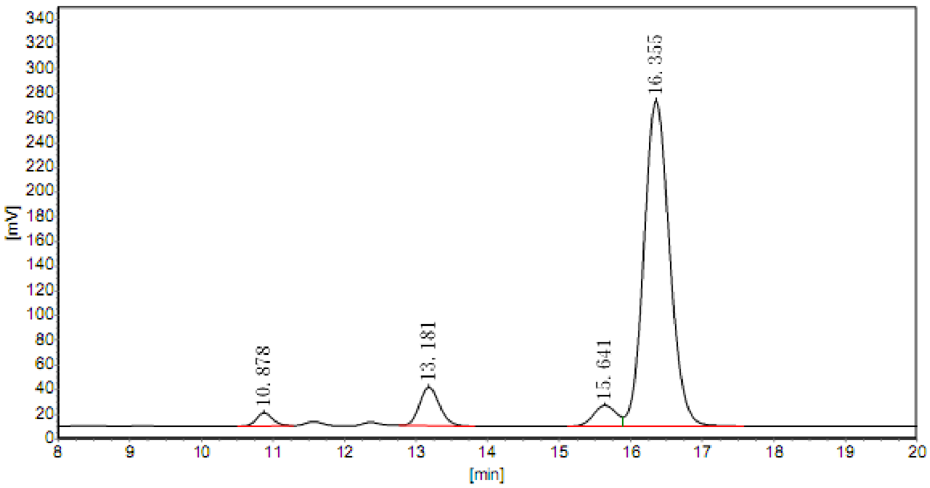

DEFAULT REPORT

Peak #	Time [min]	Height [μv]	Area [μv.s]	Area [%]
1	10.878	10296	160527	2.08023
2	13.181	30965	603691	7.82307
3	15.641	16730	368231	4.77180

4	16.355	263680	6584354	85.32490
Sum		321671	7716803	100.00000

Entry 4

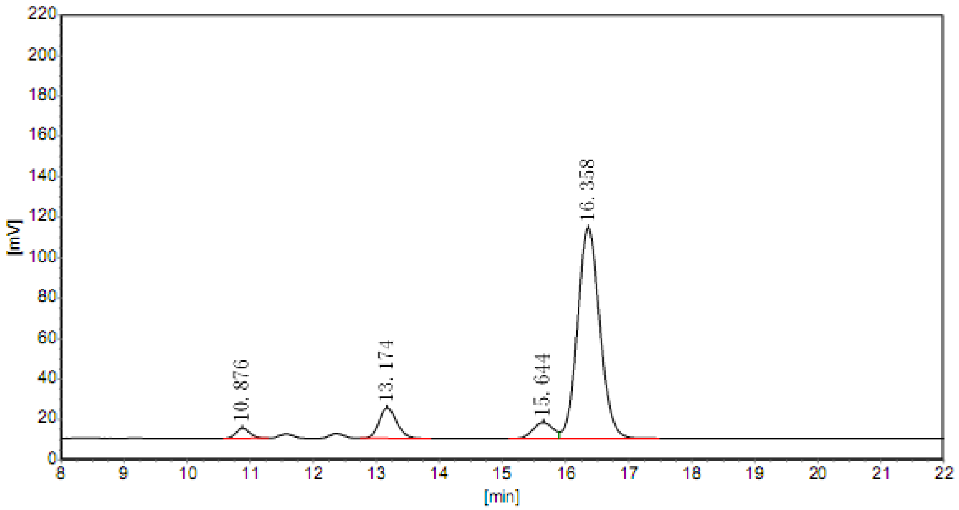

DEFAULT REPORT

Peak #	Time [min]	Height [µv]	Area [µv.s]	Area [%]
1	10.876	5071	77875	2.49880
2	13.174	14953	293861	9.42915
3	15.644	7977	176539	5.66464
4	16.358	104274	2568240	82.40742
Sum		132275	3116515	100.00001

Entry 5

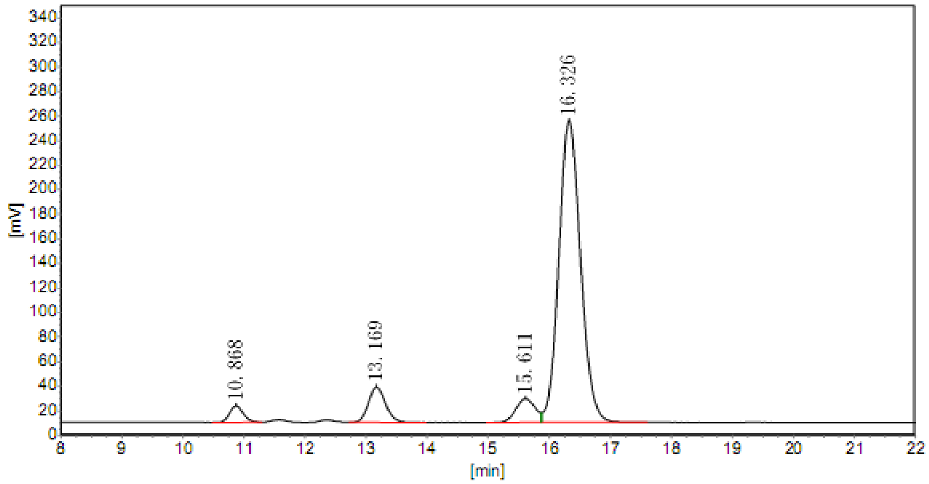

DEFAULT REPORT

Peak #	Time [min]	Height [µv]	Area [µv.s]	Area [%]
1	10.868	13285	206343	2.83828
2	13.169	28479	553169	7.60894

3	15.611	19298	426152	5.86180
4	16.326	245665	6084325	83.69098
Sum		306727	7269989	100.00000

Entry 6

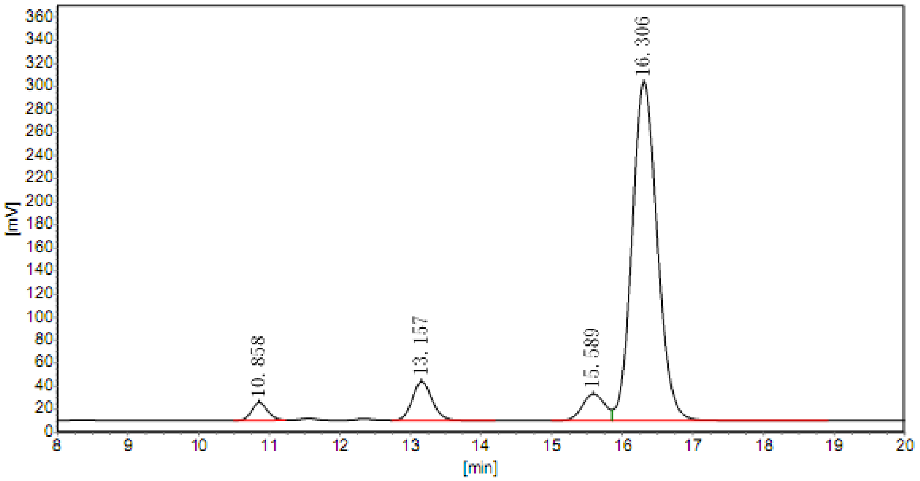

DEFAULT REPORT

Peak #	Time [min]	Height [µv]	Area [µv.s]	Area [%]
1	10.858	15134	233633	2.69958
2	13.157	33538	652285	7.53701
3	15.589	22862	503824	5.82158
4	16.306	292875	7264691	83.94184
Sum		364409	8654433	100.00001

Entry 7

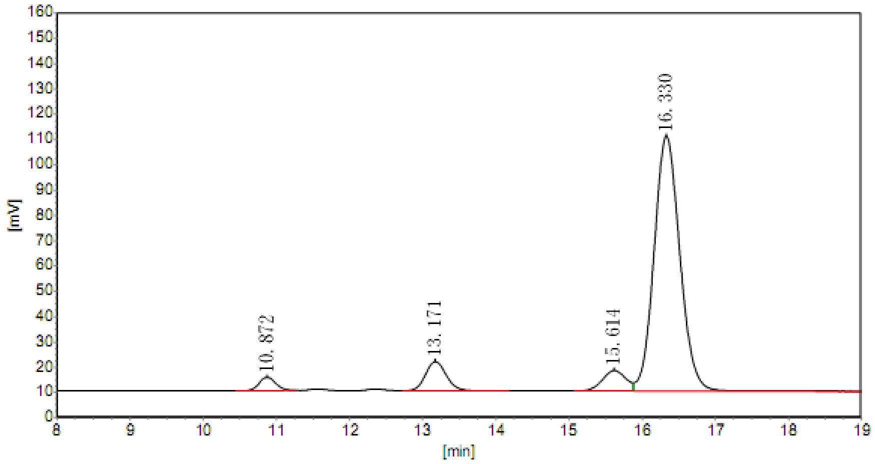

DEFAULT REPORT

Peak #	Time [min]	Height [µv]	Area [µv.s]	Area [%]
1	10.872	5366	83036	2.80277
2	13.171	11545	225065	7.59677
3	15.614	8094	178471	6.02405

4	16.330	100718	2476075	83.57642
Sum		125723	2962647	100.00001

Entry 8

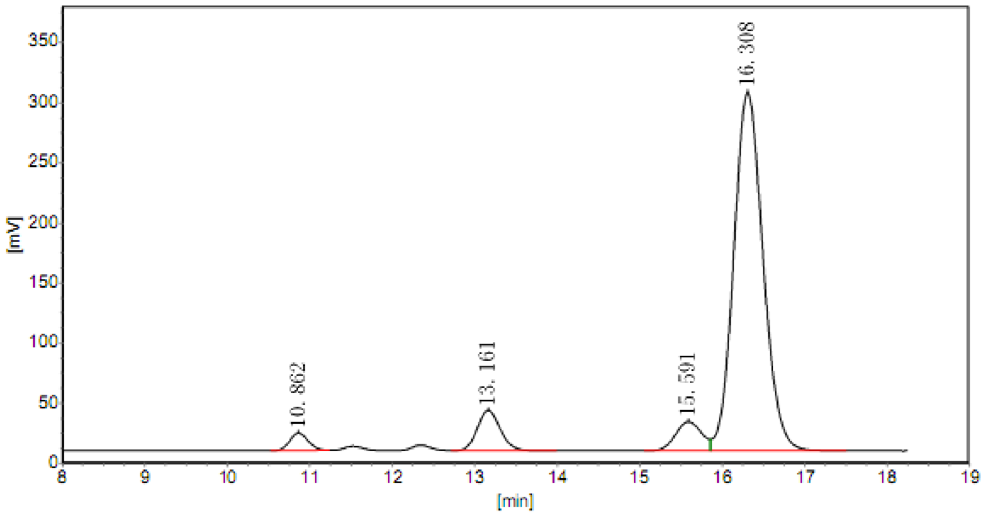

DEFAULT REPORT

Peak #	Time [min]	Height [µv]	Area [µv.s]	Area [%]
1	10.862	14485	222950	2.54360
2	13.161	33314	648792	7.40198
3	15.591	24052	529209	6.03767
4	16.308	297900	7364163	84.01675
Sum		369751	8765114	100.00000

Entry 9

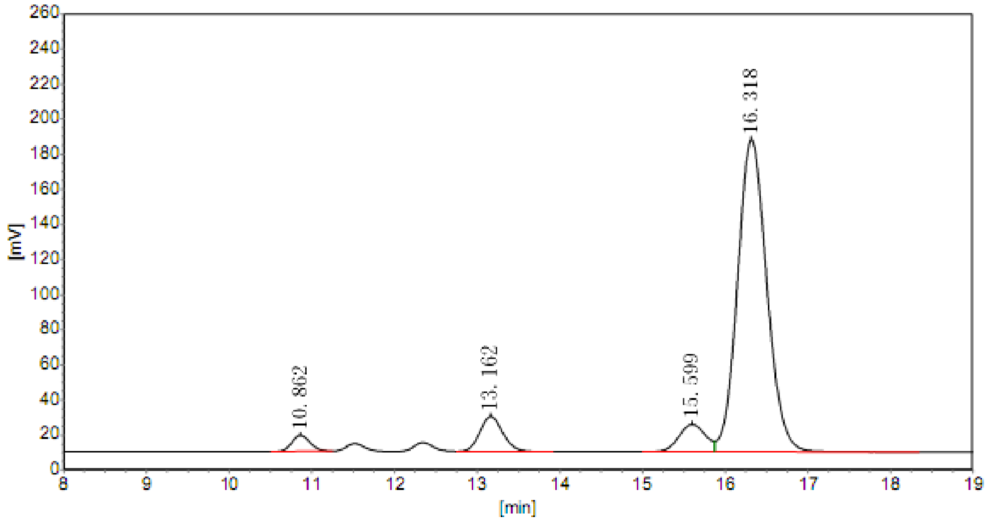

DEFAULT REPORT

Peak #	Time [min]	Height [µv]	Area [µv.s]	Area [%]
1	10.862	9171	140332	2.66522
2	13.162	19945	390045	7.40784

3	15.599	15588	347053	6.59131
4	16.318	177982	4387872	83.33562
Sum		222686	5265302	99.99999

6. References

1. Guo, Q.X., Liu, H., Guo, C., Luo, S.W., Gu, Y., Gong, L.Z. Chiral Brønsted acid-catalyzed direct asymmetric Mannich Reaction. *J. Am. Chem .Soc.* **129**, 3790-3791 (2007).

2. Zheng, X., Qian, Y.B., Wang, Y.M. 2-Pyrrolidinecarboxylic acid ionic liquid as a highly efficient organocatalyst for the asymmetric one-pot Mannich reaction. *Eur. J. Org. Chem*. **2010**, 515-522 (2010).

3. An, Y.-J., Wang, C.-C., Liu, Z.-P., Tao, J.-C. Isosteviol-proline conjugates as highly efficient amphiphilic organocatalysts for asymmetric three-component Mannich reactions in the presence of water. *Helv. Chim. Acta* **95,** 43-51 (2012).

4. Ibrahem, I., Zou, W.B., Engqvist, M., Xu, Y.M., Cordova, A. Acyclic chiral amines and amino acids as inexpensive and readily tunable catalysts for the direct asymmetric three-component Mannich reaction. *Chem. Eur. J.* **11**, 7024-7029 (2005).

5. List, B., Pojarliev, P., Biller, W.T., Martin, H.J. The proline-catalyzed direct asymmetric three-component Mannich reaction: scope, optimization and application to the highly enantioselective synthesis of 1,2-amino alcohols. *J. Am. Chem. Soc.* **124**, 827-833 (2002).
